# miR-3606-3p alleviates skin fibrosis by integratively suppressing the integrin/FAK, p-AKT/p-ERK, and TGF-β signaling cascades

**DOI:** 10.1016/j.jare.2024.11.027

**Published:** 2024-11-20

**Authors:** Yahui Chen, Yiyi Gong, Mengkun Shi, Haoxing Zhu, Yulong Tang, Delin Huang, Wei Wang, Chenyi Shi, Xueyi Xia, Ying Zhang, Jianlan Liu, Jia Huang, Mengguo Liu, Huyan Chen, Yanyun Ma, Ziyu Wang, Lei Wang, Wenzhen Tu, Yinhuan Zhao, Jinran Lin, Li Jin, Jörg HW Distler, Wenyu Wu, Jiucun Wang, Xiangguang Shi

**Affiliations:** a Department of Dermatology, Huashan Hospital and Human Phenome Institute, Fudan University, Shanghai, China; b Department of Thoracic Surgery, Huashan Hospital & Cancer Metastasis Institute, Fudan University, Shanghai, China; c State Key Laboratory of Genetic Engineering, Collaborative Innovation Center for Genetics and Development, School of Life Sciences, Fudan University, Shanghai, China; d Division of Rheumatology, Shanghai TCM-Integrated Hospital, Shanghai, China; e University Hospital Düsseldorf and Heinrich-Heine University, Düsseldorf, Germany; f Department of Dermatology, Huashan Hospital, Shanghai Institute of Dermatology, State Key Laboratory of Molecular Engineering of Polymers, Fudan University, Shanghai, China; g Department of Dermatology, Jing’an District Central Hospital, Shanghai, China; h National Clinical Research Center for Aging and Medicine, Huashan Hospital, Fudan University, Shanghai, China; i Ministry of Education Key Laboratory of Contemporary Anthropology, School of Life Sciences, and Academy for Engineering and Technology, Fudan University, Shanghai, China; j Deptartment of Allergy and Immunology, Huashan Hospital, and Research Center of Allergy and Diseases, Fudan University, Shanghai, China; k Research Unit of Dissecting the Population Genetics and Developing New Technologies for Treatment and Prevention of Skin Phenotypes and Dermatological Diseases (2019RU058), Chinese Academy of Medical Sciences, Shanghai, China

**Keywords:** Skin fibrosis, miR-3606-3p, ITGAV, GAB1, TGFBR2, Fibroblast abnormalities

## Abstract

**Introduction:**

Fibroblast abnormalities are crucial causes of skin fibrosis, including systemic sclerosis (SSc) and keloids. However, their mechanisms, including underlying microRNA regulatory mechanisms, remain elusive.

**Objectives:**

This study aimed to evaluate the roles, mechanisms, and therapeutic potential of miR-3606-3p in regulating multiple fibroblast abnormalities.

**Methods:**

The miR-3606-3p levels were evaluated in skin tissues and primary fibroblasts. RNA-seq and luciferase assays were employed to identify miR-3606-3p targets. Collagen contraction, western blotting, *in vivo* imaging, and real-time cellular analysis were used to assess fibroblast abnormalities. The therapeutic potential of miR-3606-3p was evaluated in mice.

**Results:**

MiR-3606-3p decreased in skin tissues (SSc: Fold Change (FC) =  − 2.95, P = 0.0101; keloid: FC =  − 3.42, P < 0.0001) and primary fibroblasts (SSc: FC =  − 12.74, P = 0.0278; keloid: FC =  − 2.08, P = 0.0021) from skin fibrosis patients, and negatively correlated with disease severity. Mechanistically, miR-3606-3p targeted the 3′-untranslated regions (3′-UTRs) of Integrin αV (*ITGAV*), GRB2-associated binding protein 1 (*GAB1*), and transforming growth factor beta receptor 2 (*TGFBR2*), all of these three targets increased in skin fibrosis. Simultaneously, miR-3606-3p inhibited fibroblast’s fibrogenesis, migration, inflammation, and proliferation by inhibiting ITGAV/integrin/FAK, GAB1/p-AKT/p-ERK, and TGFBR2/p-SMAD2/3 signaling. ITGAV-mediated integrin/FAK signaling unidirectionally activated the p-AKT/p-ERK and p-SMAD2/3 pathways. Knockdown of *GAB1* and *TGFRB2* reduced ITGAV-induced p-AKT/p-ERK and p-SMAD2/3 activities. MiR-3606-3p, *si-ITGAV*, *si-GAB1*, and *si-TGFBR2* exhibited significant inhibition of fibrogenesis and migration. Inflammation was primarily inhibited by si-ITGAV and si-GAB1, while proliferation was primarily inhibited by *si-TGFBR2*. Moreover*,* miR-3606-3p significantly attenuates skin fibrosis in keloid-bearing mice.

**Conclusions:**

MiR-3606-3p is downregulated in skin fibrosis. Moreover, it negatively correlates with disease severity. Functionally, miR-3606-3p inhibits fibrogenesis, migration, inflammation, and proliferation of fibroblasts. Mechanistically, miR-3606-3p inhibits *ITGAV*, *GAB1*, and *TGFBR2* by targeting their 3′-UTRs. ITGAV-, GAB1-, and TGFBR2-activated integrin/AKT/ERK/SMAD2/3 signaling induced fibroblast abnormalities. In vivo, miR-3606-3p inhibits skin fibrosis in mice. Therefore, the multi-targeting, multi-phenotypic regulatory properties of miR-3606-3p suggest its potential utility in clinical treatment.

## Highlights

• Downregulation of miR-3606-3p correlates with the disease severity of skin fibrosis.
• miR-3606-3p targets the 3′-UTRs of *ITGAV, GAB1*, and *TGFBR2* in fibroblasts.
• miR-3606-3p downregulation activates ITGAV/FAK, GAB1/AKT/ERK and TGFBR2/SMAD2/3.
• These cascades integratively promote proliferation, migration, and inflammation.
• miR-3606-3p alleviates skin fibrosis in keloid-bearing humanized mice.

## Graphical abstract

**In Brief** Current treatment options for systemic sclerosis (SSc) and keloid including surgery and corticosteroids each have their respective limitations, thus commanding the need for new methods of tackling these diseases. **Impact:** Our findings suggest that miR-3606-3p may be a novel potential target for various skin fibrosis diseases.Our findings add a new dimension to the interventional strategy for skin fibrosis by demonstrating the multiple effects of miR-3606-3p in simultaneously inhibiting fibroblast cell proliferation, inflammation, migration, and collagen deposition. Our study provides a valuable method to measure cell migration *in vivo* in a humanized keloid mouse model.

**Figure:**
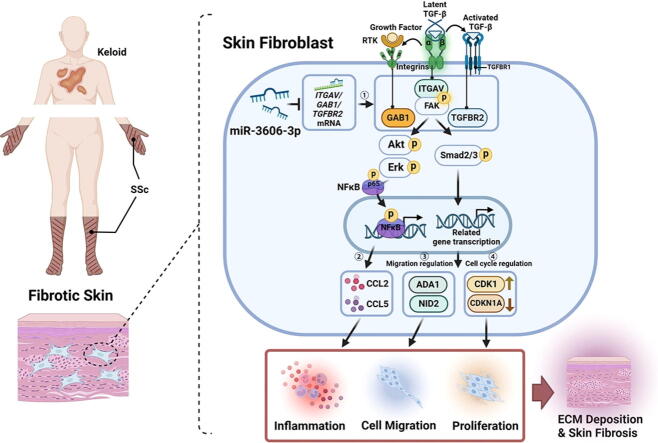

## Introduction

Skin fibrosis, represented by keloids and systemic sclerosis (SSc), is characterized by the impairment of dermal function, with structural abnormalities due to the production by fibroblasts of excessive amounts of extracellular matrix [1], [2]. Persistent pain and pruritus caused by the overgrowth of collagen bundles significantly affect the quality of patients’ lives [3], [4]. SSc is a progressive fibrotic disorder that affects 3 to 24 per 100,000 worldwide, and it has a high mortality rate when internal organs are involved, especially the lungs [5], [6]. However, due to limited available treatments, unpredictable adverse effects, and high risk of recurrence, antifibrotic strategies that aim to alleviate or revert the disease process are urgently needed [3], [7].

Fibroblast abnormalities, which play a pivotal role in the pathogenesis of skin fibrosis, include excessive collagen deposition, enhanced cell migration, heightened inflammation, and increased proliferation [8], [9], [10]. These abnormalities in fibroblasts are regulated by a variety of signaling pathways. Previous studies have demonstrated that the activities of p-AKT and p-ERK, which are mediated by receptor tyrosine kinases (RTKs), are increased in the skin fibroblasts from both keloid and SSc patients [11], [12]. In addition, transforming growth factor beta receptor II (TGFBR2)-mediated TGF-β/SMAD signaling is known to promote fibroblast proliferation, activation, migration, and extracellular matrix (ECM) deposition [13]. Integrin subunit alpha V (ITGAV) belongs to the integrin family and has been implicated in the fibrosis of various organs, including the lungs, kidneys, liver, and cardiac muscles [14], [15]. GAB1 is a member of the Gab/DOS (Daughter of Sevenless) family that acts as an adaptor of the RTK signal, thus playing a role in the regulation of p-AKT and p-ERK [16], [17]. Because ITGAV, GAB1, and TGFBR2 are upstream mediators of p-FAK, p-AKT/p-ERK, and p-SMAD signaling [18], the hyperactivity of integrin, AKT/ERK, and TGF-β in skin fibrosis may be closely related to the dysregulation of ITGAV, GAB1, and TGFBR2 levels.

MiR-3606-3p is a non-coding RNA with 21 nucleotides within the human *COL3A1* gene that shares a standard transcriptional orientation with *COL3A1*. It was first identified in 2010 by Weng-Onn Lui et al. in human cervical tissue [19]. Furthermore, in 2023, miR-3606-3p was demonstrated to be expressed in the testicular and potentially to be involved in spermatogenesis [20]. However, these studies have yet to clarify the precise mechanisms or potential targets of miR-3606-3p. Our previous studies showed that miR-3606-3p exhibits an antifibrotic effect in SSc by suppressing fibroblast proliferation and collagen deposition [21]. Indeed, fibroblasts also mediate inflammation, immunity, migration, differentiation, and other processes to promote skin fibrosis [8], [9], [10]. These results inspired us to investigate the target genes and mechanisms of miR-3606-3p in skin fibrosis for a more comprehensive understanding of its biological role, including its potential utility as a biomarker and therapeutic target.

In this study, we performed experimental assays to examine the effects of miR-3606-3p in keloid and SSc. This includes comprehensive *in vitro* assays to decipher several parallel signaling cascades that are involved in the progression of fibrosis and their varying contributions to collagen production, fibroblast proliferation, migration, fibrosis and inflammation. We further developed a mouse imaging model for detecting fibroblast migration *in vivo* and a nude mouse model for assessing skin fibrosis under scar stress. Our results reveal that miR-3606-3p attenuates integrin/FAK, AKT, ERK, and SMAD pathways by directly targeting *ITGAV*, *GAB1*, and *TGFBR2*, thus indicating that miR-3606-3p may serve as a novel target for simultaneously affecting multiple pathways that lead to diverse fibrotic diseases.

Currently, there are some solutions and important results of the other authors in relation to the experimental topic we have approached. Previous studies have identified fibroblast abnormalities in organ fibrosis, which reinforces our findings that fibroblast abnormalities, including excessive collagen deposition, migration, inflammation, and proliferation, contribute to skin fibrosis [8], [9], [10]. Methodologically, the study of cell migration is mainly limited to *in vitro* experiments. We developed a strategy to monitor fibroblasts' *in vivo* migration rate based on *in vivo* imaging technology in mice. Furthermore, we discovered that the Integrin/FAK, AKT/ERK, and TGF-β/SMAD2/3 pathways play important roles in these fibroblast abnormalities. These pathways have been reported to be aberrantly activated in skin fibrosis [11], [12]. Existing literature also suggests a regulatory interplay among Integrin/FAK, AKT/ERK, and TGF-β/SMAD2/3 pathways [22], [23], [24], [25], [26]. However, the precise nature of this regulatory network remains unclear. Our results reveal that Integrin/FAK activates AKT/ERK and TGF-β/SMAD2/3 unidirectionally, and this activation depends on GAB1 and TGFBR2, respectively. Several microRNAs have been reported to modulate fibroblast abnormalities and skin fibrosis [27], [28]. We are the first to present novel insights into miR-3606-3p's role and mechanism in regulating these abnormalities by influencing the Integrin/FAK, AKT/ERK, and TGF-β/SMAD2/3 pathways and their potential therapeutic implications. Additionally, ITGAV, GAB1, and TGFBR2 have previously been shown to be regulated by microRNAs [21], [29], [30]. For the first time, we have demonstrated that all three genes are targets of miR-3606-3p.

In our opinion about future perspectives, fibroblast abnormalities are not the result of a single phenotype or pathway dysregulation but rather a combination of multiple genes and signaling molecules. More than one phenotypic description of fibroblast dysfunction is required to fully understand the disease's pathogenesis and identify effective intervention targets. Therefore, it is necessary to find a multi-targeted, potent, and long-lasting fibrotic regulator and to elucidate its regulatory effect on fibroblast abnormalities from multiple dimensions in order to eventually intervene in skin fibrosis.

## Materials and methods

### Antibodies, reagents, and resource

**Antibodies:** Antibodies against p65 (#8242), p-p65 (#3033), SMAD2/3 (#5678), p-SMAD2/3 (#8828), ERK1/2 (#4695), p-ERK1/2 (#4370), AKT1 (#2938), p-AKT1 (#4060), NID2 (#86513S), and GAPDH (#5174) were purchased from Cell Signaling Technology (Danvers, MA, USA). Antibodies against S100A4 (ab197896), GAB1 (ab59362), ADA1 (65184S), and Collagen Type III (ab7778) were purchased from Abcam (Cambridge, MA, USA). Antibodies against α-SMA (GB111364) and Ki-67 (GB111141) were purchased from Servicebio (Wuhan, Hubei, China). Antibodies against FAK (A11131), p-FAK (AP1447), CDK1 (A11420), and CDKN1A (A1483) were purchased from ABclonal (MA, USA). Antibodies against ITGAV (sc-9969), Collagen Type I (AB758), and TGFBR2 (66636-1-Ig) were purchased from Santa Cruz Biotechnology (CA, USA), Millipore (Burlington, MA, USA), and Proteintech (Rosemont, IL, USA), respectively. **Reagents:** Lipofectamine RNAiMax (13778) and Lipofectamine 2000 (11668500) were purchased from Life Technologies (Carlsbad, CA, USA). 4-amino-6-diamino-2-phenyl indole (DAPI) (D8417) was from Sigma (St. Louis, MO, USA), and Pierce™ ECL Western (32106) was from Pierce (Waltham, MA, USA). The *si-ITGAV, si-GAB1, si-TGFBR2,* miR-3606-3p mimic*,* miR-3606-3p inhibitor*,* and NC were synthesized by Genomeditech (Shanghai, China). The fluorescence FISH probe for miR-3606-3p was purchased from Servivebio (Wuhan, Hubei, China). The *si-ADA1* (AM16708) and *si-NID2* (#4392420) siRNAs were purchased from Thermo Fisher Scientific (Waltham, USA). **Critical Commercial Assays:** Commercial assays included the Dual-Luciferase Reporter Assay System (E1910, Promega, Madison, WI, USA), Nuclear and cytoplasmic protein extraction kit (SC-003, Invent Biotechnologies, Inc., USA), Cell Contraction Assay Kit (CBA201, Cell Biolabs, CA, USA), Cell Cycle and Apoptosis Analysis Kit (40301ES50, YEASEN, Shanghai, China), Annexin V-FITC and PI Staining Solution in the Cell Cycle and Apoptosis detection kit (40302ES20, YEASEN, Shanghai, China). **Cell Lines:** HFF-1 cells were purchased from the American Type Culture Collection (SCRC-1041, ATCC). **Mice:** Balb/c nude mice were purchased from Biocytogen (Shanghai, China).

### Ethics statement

All animal experiments were operated according to the guidelines approved by the Institutional Animal Care and Use Committee of Fudan University (Approval no. FE20002). All experiments involving human patients were conducted according to the ethical policies and procedures approved by the ethics committee of the School of Life Sciences of Fudan University (Approval no. KY2023-015). All participants signed informed consent forms and were notified about the study before participating.

### Patients

The biopsy skin tissues from 22 SSc patients and 26 patients with keloids were obtained from Shanghai Huashan Hospital, Fudan University. Healthy individuals with no history of autoimmune or other skin diseases were included in the control group for comparison. The criteria developed by the American College of Rheumatology and the European League Against Rheumatism were applied to diagnose SSc patients and organ involvement. Patients with keloids were diagnosed according to the classification and evaluation criteria of the JSW Scar Scale (2015). The modified Rodnan skin thickness score (MRSS) was analyzed according to previous studies [31]. Detailed information is listed in Supplemental Table S1. This study was approved by the ethical committee of Fudan University, China.

### Construction of keloid-bearing skin fibrosis mice and treatments

Six-week-old male Balb/c nude mice were used in this study. Keloid dermal tissues were cut into 5 mm × 5 mm × 5 mm pieces of similar weight. Nude mice were anesthetized with 1.25 % tribromoethanol, a 1 cm cutaneous incision was made under the lower right shoulder blade, and keloid tissues were implanted. The keloid-bearing mice were fed under standard conditions for 14 days and then were used in subsequent experiments.

### Evaluation of the therapeutic potential of miR-3606-3p

Ten nude mice with keloids were randomly distributed into two groups. Negative Control (NC), miR-3606-3p mimic or inhibitor with 5′-Cholesteryl and 2′-O-methyl modification (3 nmol dissolved in 50 μl PBS) was injected into the keloid tissue every 3 days. After 24 days, the mice were sacrificed, and the keloid tissues were harvested. The morphology and weight of the keloid tissues were recorded.

### *In vivo* imaging migration assay

The skin fibroblast cell line HFF-1 was transfected with CY3-labeled NC, miR-3606-3p mimics or inhibitors and then was injected subcutaneously into 6 keloid-bearing mice at a symmetrical positions 1 cm away from the keloid. The mice were housed in a dark environment. Twenty-four hours later, Cy3 fluorescence was observed in anesthetized mice using In Vivo Xtreme (Bruker, Germany). The migration rate was calculated as follows: Migration rate = 100 × (1-C), where C is the fluorescence front-end distance from the center of the keloid tissue.

### Histology and immunofluorescence analysis

The collected SSc, keloid, and normal skin tissue samples were fixed in 4 % paraformaldehyde and then embedded in paraffin for hematoxylin and eosin (H&E) staining, Masson’s staining, Immunohistochemistry (IHC), fluorescence in situ hybridization (FISH), and immunofluorescence staining (IF). H&E and Masson’s staining was performed according to standard histological protocols. The fluorescence probe of miR-3606-3p for FISH was designed by Servivebio, China, and FISH was performed according to standard protocol. For IHC and IF, samples were incubated overnight at 4 °C with primary antibodies, including anti-Ki-67, anti-p65, anti-αSMA, anti-S100A4, anti-ITGAV, anti-GAB1, or anti-TGFBR2, followed by incubation with the second antibody at room temperature for 1 h. IHC sections were visualized with 3,3′-diaminobenzidine (DAB) peroxidase substrate (Servicebio, China) and counterstained with hematoxylin (Servicebio, China). Where indicated, nuclei were counterstained with DAPI (Sigma, St. Louis, MO, USA). Fluorescence confocal images were captured using an LSM 5 Pascal Laser Scanning Microscope (Zeiss, Germany).

### Fibroblast cell culture

Primary fibroblasts were obtained as previously described. Briefly, skin biopsy samples were washed in 75 % ethanol, phosphate-buffered saline (PBS), and complete Dulbecco's modified Eagle's medium (DMEM). Primary fibroblast strains were established by mincing tissues and placing them in 60 mm glass-covered dishes. The third to fifth passages of primary dermal fibroblasts were used for gene and protein detection. All fibroblast cells and HFF-1 cell lines were cultured in DMEM medium with 10 % FBS, 2 mM glutamine, and 50 mg/ml gentamicin at 37 °C in 5 % CO_2_.

### Real-time quantitative reverse transcriptase-polymerase chain reaction (qRT-PCR)

Total RNA was isolated using the HP Total RNA Kit protocol (Omega Biotech, Stamford, CT, USA). An M−MLV First Strand Kit (Life Technologies, Gaithersburg, MD, USA) was used to reverse-transcribe the RNA to cDNA using random reverse transcription (RT) primers for total mRNA or specific primers for miR-3606-3p and U6. SYBR-Green-based qRT-PCR analysis was conducted to quantify the transcriptional levels of genes, and miR-3606-3p was evaluated on a Roch-LC480 Real-Time PCR system (Roche, Switzerland). The reverse transcription and qPCR primer sequences of U6 and miR-3606-3p, and the qPCR primer sequences of mRNAs are detailed in Supplemental Table S2. Relative mRNA and miR-3606-3p levels were normalized to *β-actin* and *U6*, respectively.

### Prediction of target genes for miR-3606-3p

TargetScan and miRDB software were used to scan and improve the accuracy of predicting potential targets for miR-3606-3p. The miRNA-mRNA interactions were predicted according to the seed sequence match, contribution of multiple binding sites, site accessibility, free energy of the miRNA-mRNA duplex, local mRNA sequence, and local ALU content. Venn diagram analysis was used to further identify the overlap in predicted target genes obtained from TargetScan and miRDB and miR-3606-3p-downregulated DEGs from RNA-Seq analysis.

### Construction of recombinant plasmids containing 3′-UTRs of target genes with predicted miR-3606-3p-binding sites

Two *ITGAV* 3′-UTR fragments (Positions 1570–1577 and 1802–1808), five *GAB1* 3′-UTR fragments (Positions 2251–2257, 3122–3128, 4024–4030, 4174–4180 and 5175–5182), three *TGFBR2* 3′-UTR fragments (positions 7258–7265, 7329–7335 and 8163–8169), as well as a target gene fragment containing the predicted miR-3606-3p binding sites, were directly synthesized (GenScript Biotech, Shanghai, China) and separately cloned into pmirGLO plasmid. As controls, the seed region of the predicted miR-3606-3p target sites in the wild-type recombinant plasmids were replaced with mutational bases and defined as mutant pmirGLO-*ITGAV*-3′-UTR, mutant pmirGLO-*GAB1*-3′-UTR, and mutant pmirGLO-*TGFBR2*-3′-UTR, respectively. The sequences of the wild-type and mutant fragments are listed in Supplemental Table S3.

### Luciferase reporter assay

Fibroblasts were seeded in 24-well plates, and 800 ng/well recombinant plasmids were co-transfected with either negative control (NC) or miR-3606-3p mimic using Lipofectamine 2000 (Invitrogen). The Dual-Luciferase Reporter Assay System (Promega, Madison, WI, USA) was used to analyze the luciferase activity of each recombinant plasmid. All experiments were performed in triplicate. The relative Firefly luciferase activities were normalized to Renilla luciferase activity.

### Exogenous overexpression of miR-3606-3p by transfection of miRNA mimics in dermal fibroblasts

Dermal fibroblasts were seeded on 12-well plates at a density of 1 × 10^5^ cells per well. 24 h later, when the cells reached approximately 60–80 % confluence, miR-3606-3p overexpression assays were performed. The miR-3606-3p mimic (GenePharma, China) comprised the sequence of the human miR-3606-3p. As a control, a non-targeting small interfering RNA was used. The sequences were as follows:

miR-3606-3p: 5′-AAAAUUUCUUUCACUACUUAG-3′.

NC: 5′-UUCUCCGAACGUGUCACGUTT-3′.

The cells were transfected with 50 nM miRNA mimic and RNAiMax (Invitrogen) and were collected for RNA-seq after 24 h. All experiments were performed in triplicate.

### RNA interference

Human fibroblasts were cultured on 12-well plates at a density of 1 × 10^5^ cells per well. 24 h later, when the cells reached approximately 60–80 % confluence, the knockdown assays were performed using RNAiMax (Invitrogen) and 100 pmol of siRNA. The siRNAs of *ITGAV*, *GAB1*, *TGFBR2*, and NC were synthesized by Genomeditech (Shanghai, China). The sequences were as follows:

*si-ITGAV*: 5′-GUGCAAUCUUGUACGUAAATT-3′.

*si-GAB1*: 5′-GAGAGUGGAUUAUGUUGUUUU-3′.

*si-TGFBR2*: 5′-GATTCAAGAGTATTCTCACTT-3′.

NC: 5′-UUCUCCGAACGUGUCACGUTT-3′.

*si-ADA1* and *si-NID2* siRNAs were purchased from Thermo Fisher Scientific. The cells were collected for RNA, wound healing, and protein evaluation after 24 or 48 h.

### Nuclear and cytoplasmic protein extraction

A nuclear and cytoplasmic protein extraction kit (SC-003, Invent Biotechnologies, Inc., USA) was used for extract preparation according to the manufacturer’s instructions. When the fibroblast cells had grown to 90–100 % confluence, pre-cooled PBS was added to the cell culture plate to remove the supernatant. Cytoplasmic Extraction Buffer was then added to the cells, which were incubated for 5 min on ice. The cells were then vortexed vigorously for 15 s and centrifuged at 4 °C, maximum speed (14000–16000 × g) for 5 min. The supernatant contained total cytoplasmic protein. Next, nucleus extraction buffer was added to the precipitate, which was vortexed vigorously for 15 s, followed by 1 min incubation on ice for four rounds. Finally, the nucleoprotein was isolated after centrifugation at 14000–16000 × g for 30 s. The fractions were separated by SDS-PAGE.

### Western blot analysis

Total protein was extracted from fibroblast lysates and then fractionated by SDS-PAGE electrophoresis for 1.5 h. Two hours later, the proteins were transferred to membranes (Millipore, Billerica, MA, USA) at 300 mA. Subsequently, 5 % BSA/TBST buffer was used to block the nitrocellulose membranes for 1 h at room temperature, and then primary and secondary antibodies were incubated overnight at 4 °C or for 2h at room temperature. Finally, protein levels were detected using an ECL kit (Pierce, Rockford, IL, USA). All experiments were done in triplicate. Protein levels were normalized to GAPDH. Primary antibodies used in the analysis were as follows: anti-Collagen Type I, anti-Collagen Type III, anti-GAB1, anti-ITGAV, anti-TGFBR2, anti-p-ERK1/2, anti-ERK1/2, anti-p-AKT1, anti-AKT1, anti-p-SMAD2/3, anti-SMAD2/3, anti-p-FAK, anti-FAK, anti-p65, anti-p-p65, anti-αSMA, anti-ADA1, anti-NID2, anti-CDK1, anti-CDKN1A, and anti-GAPDH. Relative protein levels were calculated by densitometry analysis using Image J 1.8.0. Uncropped immunoblot gels are shown in the Supplemental File.

### Extracellular collagen measurement

Acid-soluble collagen in cell supernatants of cultured fibroblasts or whole skin homogenates of keloid grafts were collected to measure the total soluble collagen using a Sircol assay kit (Biocolor, Belfast, UK). The relative levels of total soluble collagens were normalized to the total protein of cell lysates, determined by BCA Assay (Beyotime, Nanjing, China). Briefly, prepared assay samples were added directly to the wells of a microplate. Then, 175 µl of Sircol Dye Reagent was added to the wells. The microplate was placed on a microplate shaker for 30 min at 300 rpm. During this time, any dye-labeled collagen formed an insoluble precipitate. After additional shaking at 1500 × g for 90 min, 250 µl of Plate Wash Reagent was added to each well, and the plates were centrifuged at 1500 × g for 30 min. Then, 200 µl of Dye Release Reagent was added to the appropriate sample microwells. The plates were placed on the microplate shaker (700 rpm) for approximately 20–30 min until a uniform coloration was observed in the highest concentration assay standard. The absorbance was measured at 540 nm using an MRX ELISA reader (Dynex Technologies).

### Collagen gel contraction assay

The Cell Contraction Assay Kit (Cell Biolabs, CA, USA) was used to construct a two-step collagen contraction model according to standard protocol. SSc or keloid primary fibroblasts were transfected with miR-3606-3p mimic, *si-ITGAV*, *si-GAB1*, or *si-TGFBR2* for 48h. The cells were then harvested and resuspend in the desired medium at 2 × 10^6^ cells/ml. The cell-collagen mixture was incubated for 1h at 37 °C, and 1 mL medium was added. After 48 h, the gel was released using a scraper, and its size was measured with Image J 1.8.0.

### Wound healing scratch assay

A scratch assay was performed to measure the effect of miR-3606-3p, *si-ITGAV*, *si-GAB1*, and *si-TGFBR2* on skin fibroblast migration. When fibroblasts reached confluence after transfection, cell scratches were induced using 200 μL tips, and the plates were washed with PBS three times. Fibroblasts were grown in DMEM containing 0.1 % FBS to interfere with cell proliferation. The maximal distance for each scratch was measured as the gaps between both sites of the scratches. Wound repairability was used to evaluate the migration rate according to the following equation: wound repair = 100 × (A − B)/A, where A is the width of the scratch at 0 h, and B is the width of the scratch at 48 h.

### RTCA assay

The cell proliferation ability was detected using the Real Time Cellular Analysis (RTCA) kit from Roche. SSc and keloid primary fibroblasts from the third to fifth passages were diluted in a complete culture medium at a concentration of 5000 cells/well and were seeded on E-Plates 16 (Roche). After reaching approximately 60–80 % confluence, the fibroblasts were transfected with miR-3606-3p mimic, *si-ITGAV*, *si-GAB1*, or *si-TGFBR2*. Over 84 h, the cell index, which denotes the percentage of well space occupied by cells, was measured at 10 min intervals.

### Flow cytometry detection of the cell cycle and apoptosis

Primary fibroblasts were cultured in 12-well plates and transfected with miR-3606-3p mimics, *si-ITGAV*, *si-GAB1*, *si-TGFBR2*, or NC for 72 h. Subsequently, 5 × 10^5^ cells were collected by trypsinization. For cell cycle detection, cell precipitates were gently mixed with 1 mL of pre-cooled 70 % ethanol and fixed overnight at 4°C. Next, the cells were collected by centrifugation at 1000 × g for 5 min. Flow cytometry detection was performed at an excitation wavelength of 488 nm using PI solution staining with the Cell Cycle and Apoptosis Analysis Kit. For apoptosis detection, the cells were incubated with binding buffer containing Annexin V/FITC and propidium iodide for 15 min in the darkness. The excitation wavelengths of flow cytometry were 488 nm and 535 nm. All results were analyzed using FlowJo 10.6.2.

### RNA sequencing

Total RNA was extracted from miR-3606-3p- or NC-treated skin fibroblast cells and cDNA libraries were constructed using the KAPA RNA HyperPrep kit (Kapa Biosystems, Wilmington, MA, USA) according to the manufacturer’s protocol. The cDNA libraries were sequenced using an Illumina HiSeq X Ten system (Illumina, USA). Kallisto and Deseq2 were used to analyze the transcriptome data and identify differentially expressed genes (DEGs; P < 0.05).

### Statistical analysis

Statistical analyses were performed using GraphPad Prism9.0 software. To compare the effect of miR-3606-3p, *si-ITGAV*, *si-GAB1*, and *si-TGFBR2* on inflammation with or without TNF-α stimulation, One-way or two-way Analysis of Variance (ANOVA), along with multiple comparison tests, were used. Two-sided Student's t-tests were performed to evaluate the therapeutic effect of miR-3606-3p. For comparisons of multiple pairs of control and treatment, multiple treatments and a single control, or multiple treatment groups, the Benjamini-Hochberg procedure, Dunnett's test, and Tukey's test were adopted accordingly. The Spearman's rank correlation coefficient was used to evaluate correlations between the two groups. P-values less than 0.05 were considered significant. Raw data was included in the “Raw data” file.

## Results

### MiR-3606-3p is decreased in skin fibrosis and is negatively correlated with disease severity

**Fig. 1:**
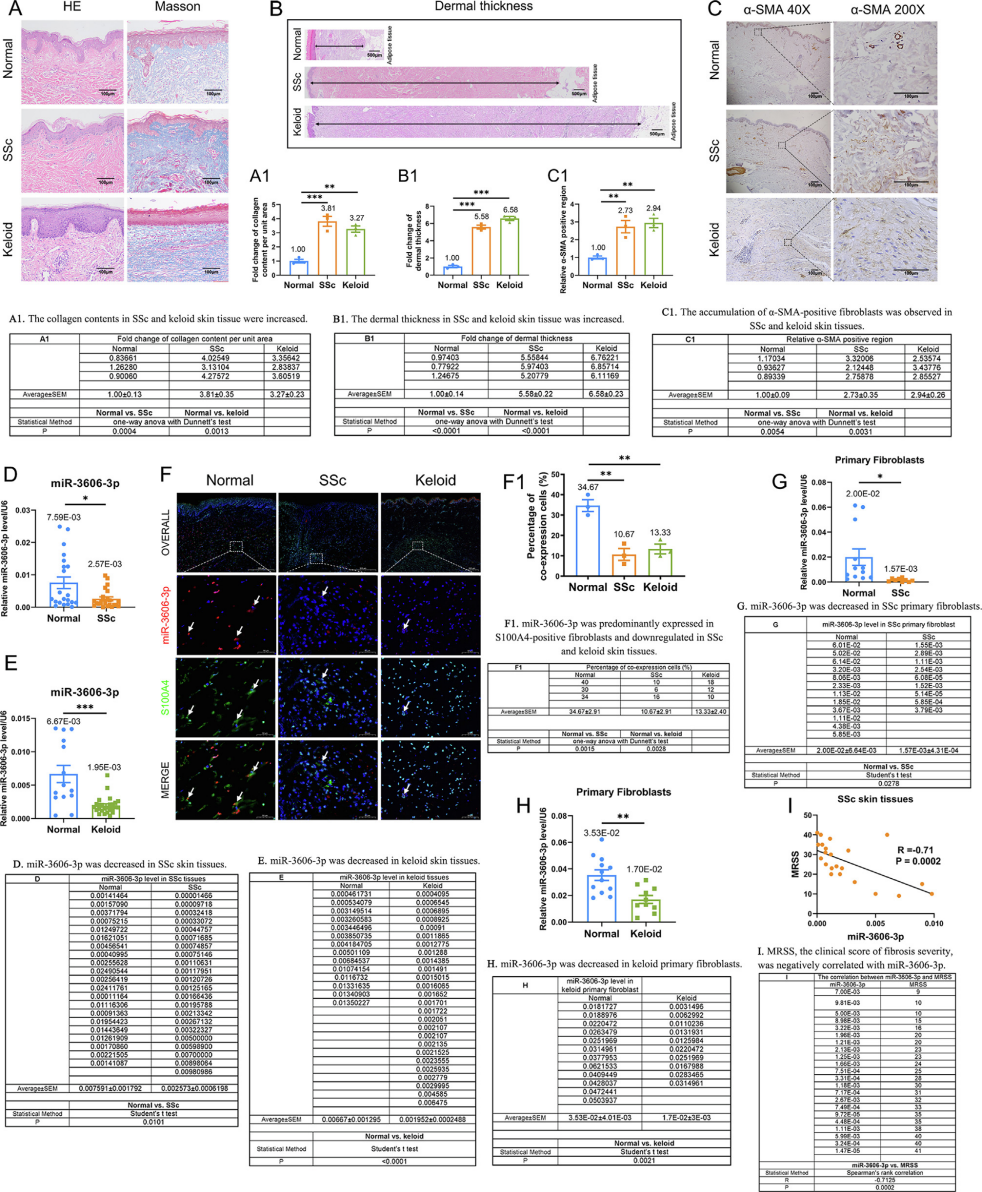
**(Relative to Stage 1). MiR-3606-3p expression in skin dermal tissues is negatively correlated with disease severity. (A-A1**) HE staining, Masson’s staining, and collagen content per unit area of normal control, SSc, and keloid skin tissues. Scale bar, 100  μm. (**B-B1**) The dermal thickness of normal control, SSc, and keloid skin tissues. Scale bar, 500  μm. (**C**) IHC staining and (**C1**) quantification showing α-SMA-positive regions in normal control, SSc, and keloid skin tissues. (**D**) Reduced miR-3606-3p levels in SSc skin tissues (22 SSc patients *vs.* 21 controls). (**E**) The expression of miR-3606-3p in skin tissues from healthy control (N = 14) and keloid patients (N = 26). (**F**) Immunofluorescence staining of miR-3606-3p and S100A4 in normal, SSc, and keloid skin tissue sections (N = 3 per group). Arrows show co-stained cells. (**F1**) The quantification of miR-3606-3p expression in double immunofluorescence staining. (**G–H**) The expression of miR-3606-3p in primary fibroblasts isolated from 9 SSc patients and 12 controls and 10 keloid patients and 12 controls. (**I**) The relationship between MRSS and miR-3606-3p in SSc skin tissues. Data in A1, B1, C1, and F1 were evaluated by one-way ANOVA (Dunnett’s test). Data in D, E, G, and H were evaluated using the Student's *t*-test. Data in I was determined by Spearman’s rank correlation coefficient. Data represented as mean ± SEM. *P < 0.05; **P < 0.01; ***P < 0.001.

Skin morphological changes, dermal structure remodeling, and collagen deposition are typical characteristics of skin fibrosis [2]. We evaluated the histopathology of SSc and keloid skin tissues compared with normal skin tissues by HE and Masson's staining and observed a 3.81- and 3.27-fold increased collagen content and 5.58- and 6.58-fold thickened dermis in SSc and keloid, respectively (Fig. 1**A–B**). In addition, immunohistochemistry staining revealed an accumulation of α-SMA-positive fibroblasts in SSc and keloid skin tissues (FC = 2.73 and 2.94, respectively) (Fig. 1**C**). These results confirm that the hyperactivation of fibroblasts and collagen deposition are common characteristics of skin fibrosis. To detect the levels of miR-3606-3p in skin fibrosis, we isolated total RNA from skin tissue homogenates and found the expression of miR-3606-3p significantly decreased in SSc (66.1 % decrease, P = 0.0101; 22 SSc patients *vs.* 21 controls) (Fig. 1**D**). Moreover, the miR-3606-3p expression was also downregulated by 70.7 % in keloid skin tissues (P < 0.0001; 26 keloid patients *vs.* 14 controls) (Fig. 1**E**).

To determine whether fibroblasts are responsible for the decreased miR-3606-3p levels in skin homogenates, we performed immunofluorescence staining. The results show that miR-3606-3p was predominantly expressed in fibroblasts. Moreover, the miR-3606-3p expression was decreased in S100A4-positive fibroblasts of SSc and keloid skin tissues compared to normal skin (Fig. 1**F**). Additionally, miR-3606-3p levels in fibroblasts from SSc skin tissues compared to controls were decreased by 92.2 % (P = 0.0278; 9 SSc patients *vs.* 12 controls) (Fig. 1**G**). Consistently, miR-3606-3p levels were significantly decreased in isolated primary fibroblasts from keloid patients compared to normal controls (P = 0.0021, 52.2 % decrease; 10 keloid patients *vs.* 12 controls) (Fig. 1**H**).

To evaluate the relationship between miR-3606-3p levels and the severity of skin fibrosis, we used Spearman’s correlation analysis. MRSS, the clinical index to evaluate skin fibrosis severity, was negatively correlated with miR-3606-3p (P = 0.0002, R =  − 0.71) (Fig. 1**I**). These results suggest that the low expression of miR-3606-3p may contribute to the severity of skin fibrosis.

### MiR-3606-3p induces a distinct gene profile in fibroblasts and reduces ITGAV, GAB1, and TGFBR2 expression by directly targeting their 3′-UTRs

**Fig. 2:**
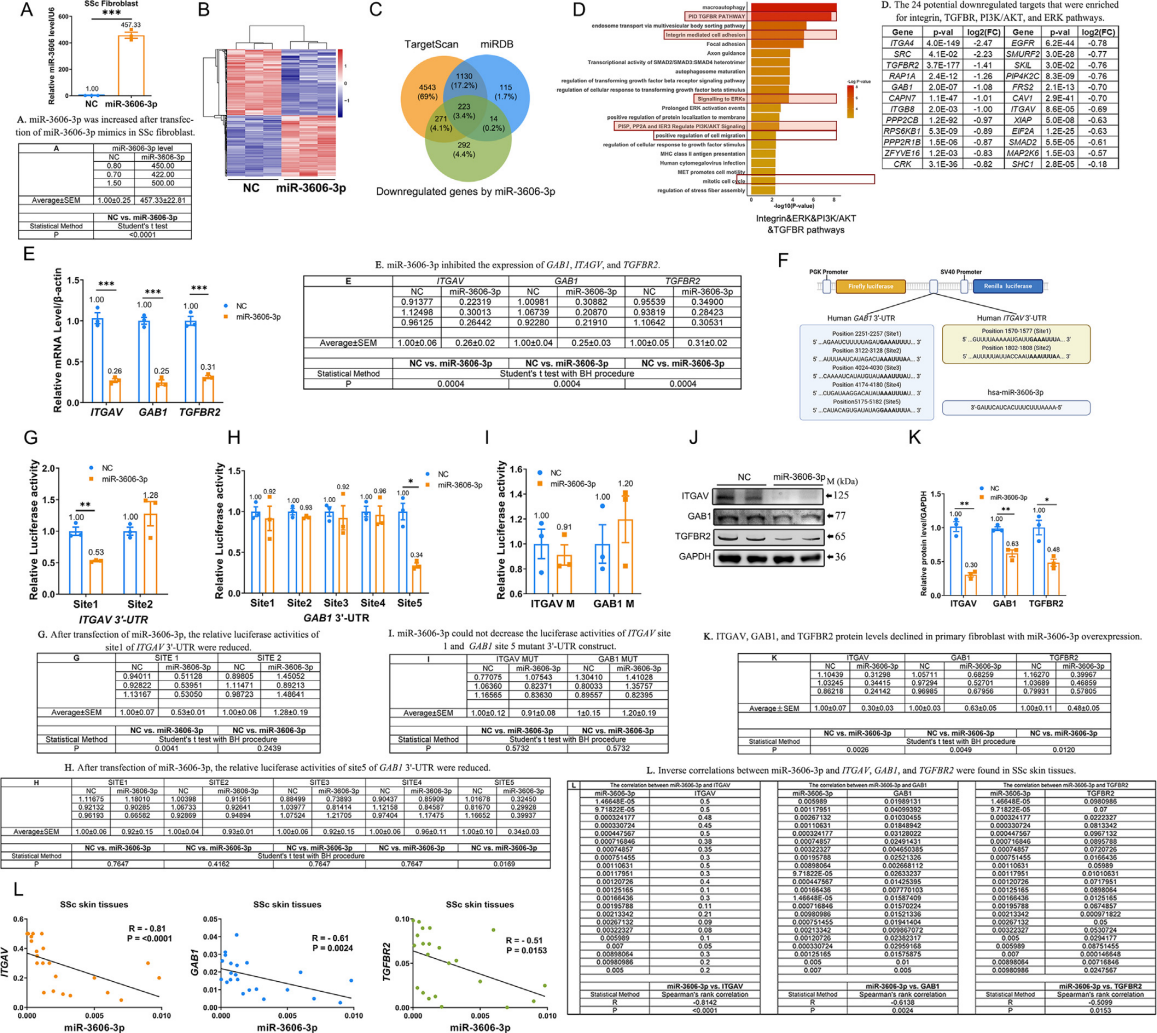
**(Relative to Stage 2). Identification of DEGs in miR-3606-3p-overexpressing fibroblasts and verification of the miR-3606-3p-mediated decrease of ITGAV, GAB1, and TGFBR2.** (**A**) The miR-3606-3p expression in fibroblasts transfected with NC (negative control) or miR-3606-3p mimic. (**B**) Heatmap of DEGs between miR-3606-3p- and NC-transfected fibroblasts. (**C**) Venn diagram analysis of decreased DEGs (P < 0.05) and genes predicted by TargetScan and miRDB to be potential miR-3606-3p targets. (**D**) GO and KEGG analysis of DEGs predicted to be candidate target genes of miR-3606-3p. (**E**) mRNA expression of *ITGAV, GAB1*, and *TGFBR2* in primary fibroblasts. The experiment was performed in triplicate. (**F**) Potential miR-3606-3p binding sites in *ITGAV* and *GAB1* 3′-UTRs. (**G**) Luciferase assay of two-potential wild-type *ITGAV* 3′-UTR reporter genes in primary fibroblasts. (**H**) Luciferase assay of five-potential wild-type *GAB1* 3′-UTR reporter genes in primary fibroblasts. (**I**) Luciferase assay of *ITGAV* and *GAB1* mutant constructs in fibroblast cells. (**J–K**) Protein expression of ITGAV, GAB1, and TGFBR2 in primary fibroblasts. The experiment was performed in triplicate. (**L**) Correlations between miR-3606-3p and *ITGAV, GAB1*, or *TGFBR2* (N = 22). Data in A were evaluated using the Student's *t*-test. Data in E, G, H, I, and K were evaluated by Student's *t*-test and corrected using the Benjamini-Hochberg procedure. Data in L were evaluated by Spearman’s rank correlation coefficient. Data represented as mean ± SEM. *P < 0.05; **P < 0.01; ***P < 0.001.

To understand the role of miR-3606-3p in skin fibroblasts, we exogenously overexpressed miR-3606-3p and performed an integrative analysis of the DEGs (|Fold change| ≥ 0.5; q-value < 0.05). As shown in Fig. 2**A–B**, the heatmap of the gene expression profile was distinct between the negative control (NC) group and the miR-3606-3p overexpression group. A total of 4,005 DEGs were identified, including 2,115 downregulated genes. To further clarify whether the downregulated DEGs were direct targets of miR-3606-3p, we used TargetScan and miRDB to predict target genes among the top 800 downregulated DEGs. Venn diagram analysis showed that 508 genes were both downregulated by miR-3606-3p and predicted miR-3606-3p targets (Fig. 2**C**). Furthermore, GO and KEGG orthologs showed the DEGs were major divided into TGF-β, focal adhesion, ERK, and PI3K/AKT pathways, and these DEGs largely contributed to integrin-mediated cell adhesion, immune response, cell migration, mitotic cell cycle, and so on (Fig. 2**D**). Notably, this included 24 potential downregulated targets that were enriched for integrin, TGFBR, PI3K/AKT, and ERK pathways. For additional confirmation, we performed qRT-PCR, which validated the downregulation of *ITGAV*, *GAB1*, *TGFBR2*, *PPP2CB*, and *SRC* by miR-3606-3p in fibroblasts (Fold Change > 3, P < 0.01) (Fig. 2**E and** Supplemental Fig. S1**A**). These results indicate that the antifibrotic effect of miR-3606-3p is multi-pathway and biology process-dependent.

To identify the binding sites of miR-3606-3p for these potential target genes, we performed *in silico* prediction and luciferase assays. According to TargetScan and miRDB software programs, five sites within the 3-UTR of *GAB1* (site 1: 2251–2257, site 2: 3122–3128, site 3: 4024–4030, site 4: 4174–4180, and site 5: 5175–5182), two sites within the 3-UTR of *ITGAV* (site 1: 1570–1577, site 2: 1802–1808) and 3 sites within the 3-UTR of *TGFBR2* (site 1: 7258–7265; site 2: 7329–7335; site 3: 8163–8169) were predicted to bind miR-3606-3p, further supporting our hypothesis that *ITGAV*, *GAB1*, and *TGFBR2* could be candidate targets for miR-3606-3p. Each of these potential binding sites was ligated into pmirGLO plasmids (Fig. 2**F and** Supplemental Fig. S1**B**), which were co-transfected with miR-3606-3p or NC into primary dermal fibroblasts. After transfection of miR-3606-3p, the relative luciferase activities of site1 of *ITGAV* 3′-UTR (47 % decrease, P = 0.0041), site5 of *GAB1* 3′-UTR (66 % decrease, P = 0.0169), and site3 of *TGFBR2* 3′-UTR (64 % decrease, P = 0.0023) were significantly reduced, while no significant differences were detected for the other sites (Fig. 2**G–H and** Supplemental Fig. S1**C**). To exclude the possibility of nonspecific binding, we also constructed three recombinant plasmids containing the *ITGAV* 3′-UTR mutant site1, *GAB1* 3′-UTR mutant site5, and *TGFBR2* 3′-UTR mutant site3. Luciferase assays confirmed that miR-3606-3p could not decrease the luciferase activities of these mutant 3′-UTR constructs (Fig. 2**I and** Supplemental Fig. S1**D**), thus verifying the sequence specificity of its targeting activity. As further verification, two other genes, *PPP2CB* and *SRC*, could not specifically bind to miR-3606-3p (Supplemental Fig. S1**E**). Moreover, the effect of miR-3606-3p in inducing a significant decrease in ITGAV, GAB1, and TGFBR2 was confirmed at the protein level (Fig. 2**J–K**). Additionally, inverse correlations between miR-3606-3p and *ITGAV*, *GAB1*, and *TGFBR2* were observed in SSc skin tissues (P < 0.0001, R =  − 0.81; P = 0.0024, R =  − 0.61 and P = 0.0153, R =  − 0.51, respectively) (Fig. 2**L**). These results suggest that miR-3606-3p may be involved in regulating multiple processes in both SSc and keloids and reveal three novel target genes that could potentially mediate its activity.

## ITGAV, GAB1, and TGFBR2 are increased in fibroblasts of SSc and keloids and show a positive correlation with disease severity

**Fig. 3:**
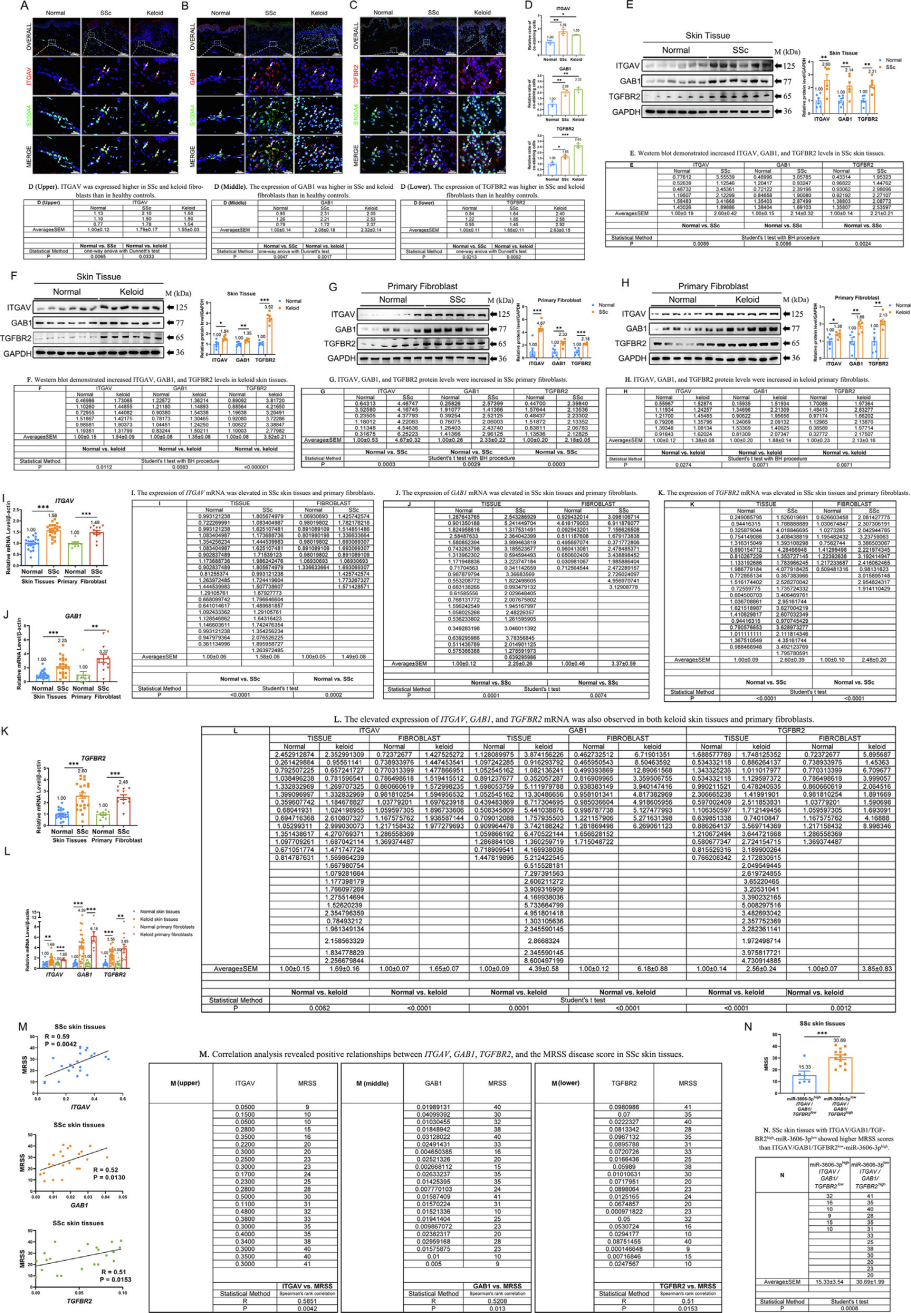
**(Relative to Stage 2). The elevated levels of ITGAV, GAB1, and TGFBR2 mediate disease severity in SSc and keloids.** (**A–C**) Skin tissue immunofluorescence staining and (**D**) quantification of healthy control, SSc, and keloids. Arrows show co-stained cells. RFI: Relative fluorescence intensity. (**E–H**) Western blot analysis of ITGAV, GAB1, and TGFBR2 protein levels in normal, SSc, and keloid skin tissues (**E–F**) and primary fibroblasts (**G–H**), calculated by densitometry (6 SSc patients *vs.* 6 controls and 6 keloid patients *vs.* 6 controls). (**I–L**) Increased mRNA levels of *ITGAV, GAB1,* and *TGFBR2* in SSc tissues (I-K) (22 SSc patients *vs.* 21 controls), primary fibroblasts (9 SSc patients *vs.* 12 controls), keloid tissues (L) (26 keloid patients *vs.* 14 controls), and primary fibroblasts (10 keloid patients *vs.*12 controls). (**M**) The relationship between *ITGAV*, *GAB1*, *TGFBR2,* and MRSS in SSc tissues (N = 22). (**N**) MRSS of *ITGAV/GAB1/TGFBR2*^low^-miR-3606-3p^high^ (N = 6) and *ITGAV/GAB1/TGFBR2*^high^-miR-3606-3p^low^ SSc skin tissues (N = 13). Data in D were evaluated by one-way ANOVA (Dunnett’s test). Data in E- H were evaluated using the Student’s *t* test and corrected using the Benjamini-Hochberg procedure. Data in I-L and N were evaluated using the Student's *t*-test. Data in M was evaluated by Spearman’s rank correlation coefficient. Data represented as mean ± SEM. *P < 0.05; **P < 0.01; ***P < 0.001.

To determine whether the three identified miR-3606-3p target genes are aberrantly expressed in skin fibrosis, we measured their mRNA and protein expression in skin tissues and primary fibroblasts. Dual immunofluorescence staining confirmed the higher expression of ITGAV, GAB1, and TGFBR2 in SSc and keloid fibroblasts as compared to healthy controls (Fig. 3**A–D**). Consistently, increased levels of ITGAV, GAB1, and TGFBR2 were demonstrated in SSc (2.60-, 2.14, and 2.21-fold, respectively) and keloid skin tissues (1.54-, 1.35, and 3.52-fold, respectively) (Fig. 3**E–F**) and primary fibroblasts (SSc: 4.67-, 2.33, and 2.18-fold, respectively; keloid: 1.38-, 1.88, and 2.13-fold, respectively) (Fig. 3**G–H**) by western blot analysis. Moreover, elevated expression of *ITGAV*, *GAB1*, and *TGFBR2* mRNA was observed in SSc skin tissues and fibroblasts (Fig. 3**I–K**) and then validated in both keloid skin tissues and primary fibroblasts (Fig. 3**L**). Correlation analysis revealed positive relationships between *ITGAV, GAB1*, *TGFBR2*, and the MRSS disease score in SSc skin tissues (P = 0.0042, R = 0.59, P = 0.013, R = 0.52 and P = 0.0153, R = 0.51, respectively) (Fig. 3**M)**. Furthermore, SSc skin tissues with *ITGAV*/*GAB1*/*TGFBR2*^high^-miR-3606-3p^low^ showed two-fold higher MRSS scores than *ITGAV*/*GAB1*/*TGFBR2*^low^-miR-3606-3p^high^ (P = 0.0008) (Fig. 3**N**), indicating that the negative correlation between miR-3606-3p and TGFBR2 is closely related to the severity of SSc. Collectively, our results indicate that the decreased miR-3606-3p and increased ITGAV, GAB1, and TGFBR2 in skin tissues and fibroblasts may be involved in the processes of both SSc and keloid formation.

### MiR-3606-3p inhibits ITGAV/FAK, GAB1/AKT/ERK, and TGFBR2/SMAD2/3 pathways to suppress collagen synthesis

**Fig. 4:**
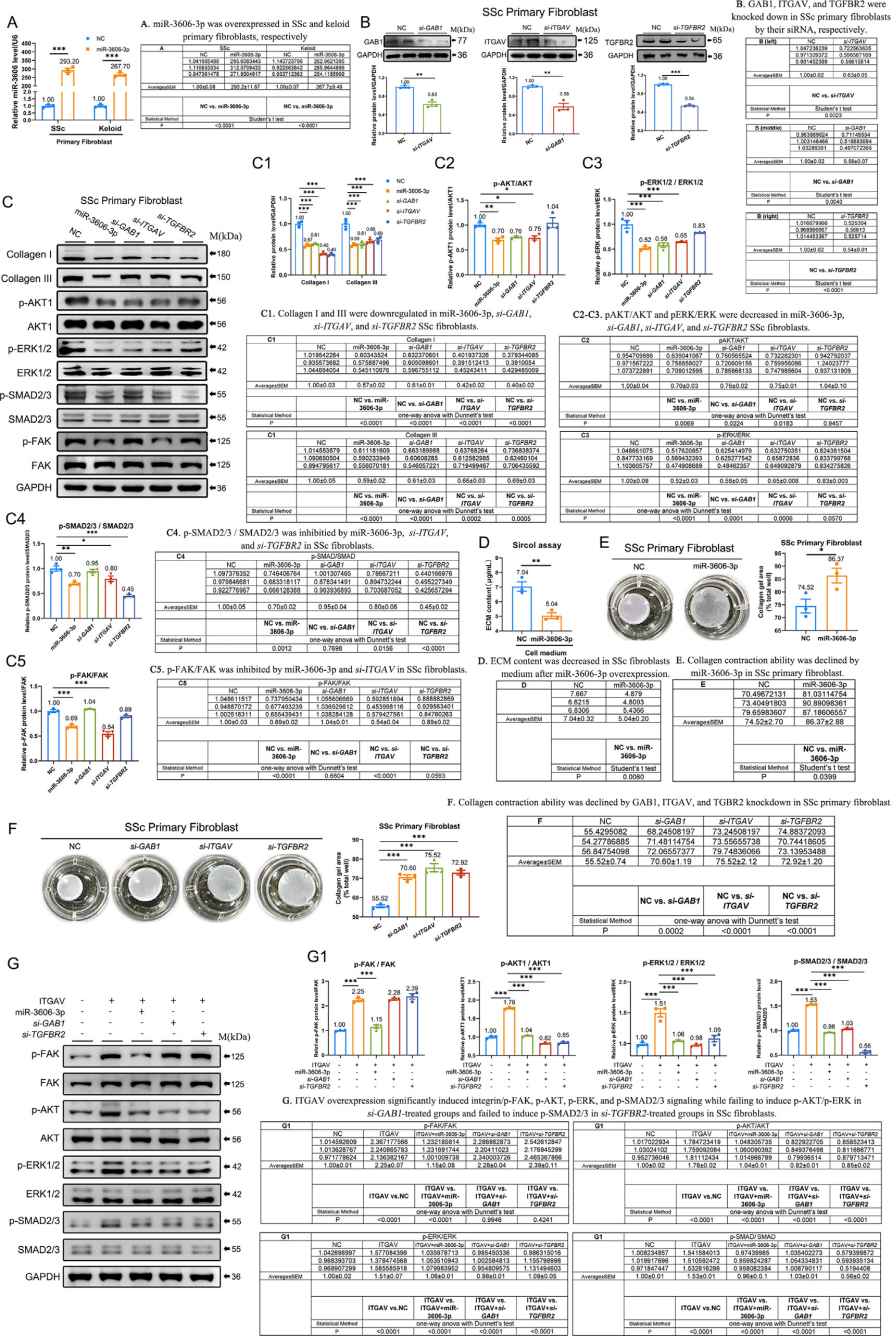
**(Relative to Stage 3). Overexpression of miR-3606-3p and knockdown of ITGAV, GAB1, and TGFBR2 suppress collagen deposition by inhibiting integrin/FAK, AKT/ERK, and SMAD2/3 pathways.** (**A**) The overexpression of miR-3606-3p in SSc and keloid primary fibroblasts. (**B**) Western blot analysis of *si-GAB1*, *si-ITGAV,* and *si-TGFBR2* in SSc primary fibroblasts. (**C**) Detection and (**C1–C5**) quantification of type I collagen, type III collagen, p-AKT, AKT, p-ERK1/2, ERK1/2, p-SMAD2/3, SMAD2/3, p-FAK, and FAK levels in SSc primary fibroblasts with NC, miR-3606-3p mimic, *si-GAB1, si-ITGAV*, or *si-TGFBR2*. (**D**) Sircol assay of SSc fibroblasts transfected with NC and miR-3606-3p. (**E–F**) Collagen contraction assay of SSc fibroblasts transfected with miR-3606-3p, *si-GAB1*, *si-ITGAV*, and *si-TGFBR2*. (**G**) Western blot analysis and (**G1**) quantification of miR-3606-3p, *si-GAB1,* and *si-TGFBR2* with ITGAV-overexpression in SSc primary fibroblasts. The experiments were repeated in triplicate for A-G. Data in A, B, D, and E were evaluated using the Student's *t*-test. Data in C1-C5, F, and G1 were evaluated by one-way ANOVA (Dunnett’s test). Data represented as mean ± SEM. *P < 0.05; **P < 0.01; ***P < 0.001.

Given that ITGAV, GAB1, and TGFBR2 belong to the integrin/FAK, AKT/ERK, and TGF-β/SMAD2/3 signaling pathways, respectively, we wondered if miR-3606-3p could downregulate collagen production by inhibiting these pathways in SSc and keloid fibroblasts. To verify these hypotheses, we overexpressed miR-3606-3p in SSc and keloid primary fibroblasts, respectively (Fig. 4**A**). Accordingly, we designed the specific siRNAs to interfere with *GAB1*, *ITGAV*, and *TGFBR2* and found that their protein levels were effectively suppressed (Fig. 4**B**). Western blots analysis showed that transfection of SSc primary fibroblasts with miR-3606-3p mimics significantly reduced the protein levels of type I collagen, type III collagen, integrin/p-FAK, p-AKT, p-ERK1/2, and p-SMAD2/3 (43 %, 41 %, 31 %, 30 %, 48 %, and 30 % decrease, respectively) (Fig. 4**C,** Fig. 4**C1–C5**), and validated in keloid primary fibroblasts (61 %, 52 %, 36 %, 53 %, 41 %, and 54 % decrease, respectively) (Supplemental Fig. S2**A-B**), illustrating that the anti-collagen-producing ability of miR-3606-3p was integrin/FAK, AKT/ERK and TGF-β/SMAD2/3 signaling-dependent. Additionally, the sircol assay and collagen contraction assay further revealed an anti-collagen-producing ability of miR-3606-3p in both SSc and keloid fibroblasts (Fig. 4**D–E and** Supplemental Fig. S2**C-D**). We next set out to further analyze the expression of collagen, p-FAK, p-AKT, p-ERK1/2, and p-SMAD2/3 in response to GAB1, ITGAV, and TGFBR2 downregulation in the fibroblasts. Western blot revealed siRNA-mediated knockdown of *ITGAV* decreased collagens, p-FAK, p-AKT1, p-ERK1/2, and p-SMAD2/3, similar to those obtained via miR-3606-3p overexpression in the SSc and keloid fibroblasts. The knockdown of GAB1 and TGFBR2 significantly suppressed collagens and the phosphorylation of ERK/AKT and SMAD2/3 as well (Fig. 4**C and** Supplemental Fig. S2**B**). Collagen contraction assay further revealed an anti-collagen-producing ability of *si-GAB1, si-ITGAV,* and *si-TGFBR2* in both SSc and keloid fibroblasts (Fig. 4**F and** Supplemental Fig. S2**E**).

To gain additional insight into the crosstalk between these pathways, we examined the interaction between these three gene-mediated signaling pathways. Results showed that *si-ITGAV* inhibited p-FAK and decreased p-AKT/ERK and p-SMAD2/3 levels. However, neither *si-GAB1* nor *si-TGFBR2* showed significant changes in integrin/p-FAK levels, suggesting that ITGAV-mediated integrin/FAK signaling is essential and unidirectional for activating downstream AKT/ERK and SMAD2/3 signaling **(**Fig. 4**C)**. To further investigate the role of GAB1 and TGFBR2 in integrin-mediated activation of AKT/ERK and SMAD2/3 pathways, we overexpressed ITGAV in SSc and keloid fibroblasts. Western blot analysis revealed that ITGAV overexpression significantly induced integrin/p-FAK, p-AKT, p-ERK, and p-SMAD2/3 signaling boh in SSc fibroblasts (2.25-fold, 1.78-fold, 1.51-fold, and 1.53-fold increase, respectively) and keloid fibroblasts (1.97-fold, 2.17-fold, 2.17-fold, and 2.42-fold increase, respectively), all of which were inhibited by miR-3606-3p **(**Fig. 4**G-G1 and** Supplemental Fig. S2**F-F1)**. In contrast, ITGAV overexpression failed to induce p-AKT/p-ERK in *si-GAB1*-treated groups, suggesting that integrin-mediated p-AKT/p-ERK is GAB1-dependent **(**Fig. 4**G-G1 and** Supplemental Fig. S2**F-F1)**. Meanwhile, ITGAV failed to induce p-SMAD2/3 in *si-TGFBR2*-treated groups, suggesting that integrin-mediated p-SMAD2/3 was TGFBR2 dependent **(**Fig. 4**G-G1 and** Supplemental Fig. S2**F-F1)**.

Collectively, our findings demonstrated the antifibrotic role of miR-3606-3p by inhibiting integrin/FAK, AKT, ERK1/2, and SMAD2/3 phosphorylation. *ITGAV, GAB1*, and *TGFBR2* are new targets for miR-3606-3p-induced collagen suppression.

### MiR-3606-3p suppresses the expression of chemokine and cytokine genes by inhibiting ITGAV- and GAB1-induced NF-κB signaling in fibroblasts

**Fig. 5:**
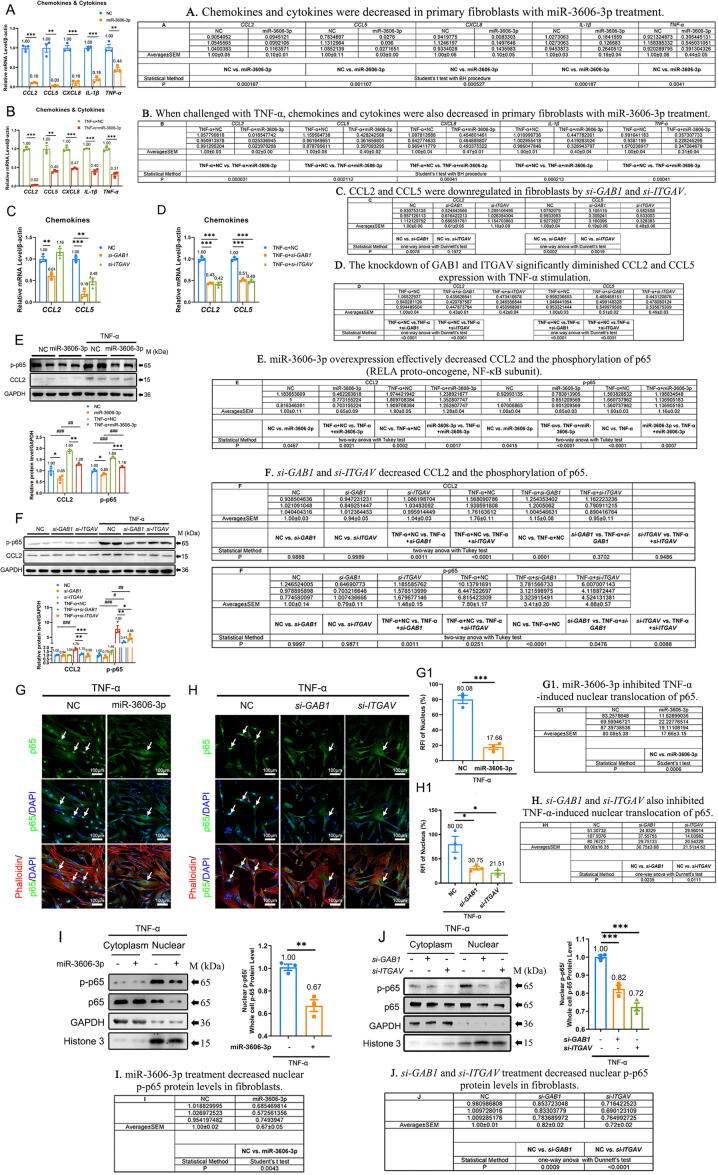
**(Relative to Stage 3). MiR-3606-3p overexpression, *si-GAB1,* and *si-ITGAV* suppress the expression of inflammatory factors and NF-κB activity.** (**A-B**) Downregulated chemokines and cytokines in miR-3606-3p overexpressing and TNF-α treated fibroblasts. (**C-D**) The effects of *GAB1* siRNA and *ITGAV* siRNA on CCL2 and CCL5 expression in fibroblasts with or without TNF-α treatment. (**E**) Detection of p-p65 and CCL2 levels by western blotting and quantification in fibroblasts transfected with miR-3606-3p mimic, with and without TNF-α treatment. (**F**) p-p65 and CCL2 protein levels in GAB1 and ITGAV knockdown fibroblasts with or without TNF-α treatment. (**G–H**) The immunofluorescence of p65 translocation to the nucleus in miR-3606-3p mimic *si-GAB1* and *si-ITGAV* fibroblasts with TNF-α stimulation. Cells were stained with TRITC conjugated phalloidin (cytoplasm) and DAPI (nuclear). Scale bar, 100 μm. (**G1–H1**) Quantification of p65 translocation. RFI: Relative fluorescence intensity. (**I–J**) Cytoplasmic and nuclear proteins were separated for p-p65 and p65 detection in miR-3606-3p mimic, *si-GAB1*, and *si-ITGAV* fibroblasts with TNF-α stimulation. All experiments were repeated in triplicate. Data represented as mean ± SEM. Data in A-B were evaluated using the Student's *t*-test and corrected using the Benjamini-Hochberg procedure. Data in C-D, H1, and J were evaluated by one-way ANOVA (Dunnett’s test). Data in E-F were evaluated by two-way ANOVA (Tukey test). Data in G1 and I were evaluated by Student's *t*-test. *P < 0.05, **P < 0.01, ***P < 0.001 within different treatment groups. #P < 0.05, ##P < 0.01, ###P < 0.001 within *vs.* without TNF-α stimulation.

Studies have demonstrated that AKT and ERK pathways play important roles in cytokine and chemokine secretion via induction of NF-κB activation [32]. Furthermore, NF-κB-mediated release of CCL2, CCL5, TNF-α, IL-1β, and CXCL8 is known to activate fibroblasts [10]. We then found a decrease in chemokines and cytokines with miR-3606-3p treatment, suggesting that miR-3606-3p could suppress inflammation in fibroblasts (Fig. 5**A and** Supplemental Fig. S3**A-B**). Interestingly, when challenged with TNF-α, a strong inflammatory inducer, miR-3606-3p could still significantly downregulate the mRNA levels of chemokines *CCL2*, *CCL5*, and *CXCL8*, and inflammatory cytokines *IL-1β* and *TNF-α* (Fig. 5**B**). These results suggest that miR-3606-3p is a critical inhibitor of fibroblast inflammation in skin fibrosis. Consistently, the knockdown of GAB1 and ITGAV significantly diminished *CCL2* and *CCL5* expression with or without TNF-α stimulation (Fig. 5**C–D**). In contrast, the expression of *CXCL8*, *IL-1β*, and *TNF-α* was not inhibited by *si-GAB1* or *si-ITGAV* with or without TNF-α (Supplemental Fig. S3**C**), suggesting that the effect of GAB1 and ITGAV are specific to CCL2 and CCL5. We also evaluated the effect of *si-TGBFR2* on the expression of these cytokines and chemokines in fibroblasts. The results demonstrate a slight change in the *si-TGFBR2* group with or without TNF-α, which indicates that the inhibitory effect of miR-3606-3p on cytokines and chemokines is primarily achieved by GAB1 and ITGAV suppression rather than TGFBR2 (Supplemental Fig. S3**D–E**).

To evaluate the role of NF-κB in miR-3606-3p-mediated cytokine and chemokine production, we performed western blot analysis. The results demonstrated that miR-3606-3p overexpression, *si-GAB1*, and *si-ITGAV* each effectively decreased CCL2 and the phosphorylation of p65 (RELA proto-oncogene, NF-κB subunit) (Fig. 5**E–F**). Consistently, miR-3606-3p, *si-GAB1*, and *si-ITGAV* each also inhibited TNF-α-induced nuclear translocation of p65 (77.9 %, 61.6 %, and 73.1 % decrease, respectively) (Fig. 5**G–H**), which was accompanied by decreased nuclear p-p65 protein levels (Fig. 5**I–J**). Collectively, these findings suggest that miR-3606-3p could serve as a novel anti-inflammatory miRNA by suppressing the synthesis of selected chemokines or cytokines, with the anti-inflammatory role of miR-3606-3p in fibroblasts explained at least partly via the reduction of GAB1 and ITGAV expression.

### Overexpression of miR-3606-3p and knockdown of ITGAV, GAB1, and TGFBR2 inhibit the migration of skin fibroblasts

**Fig. 6:**
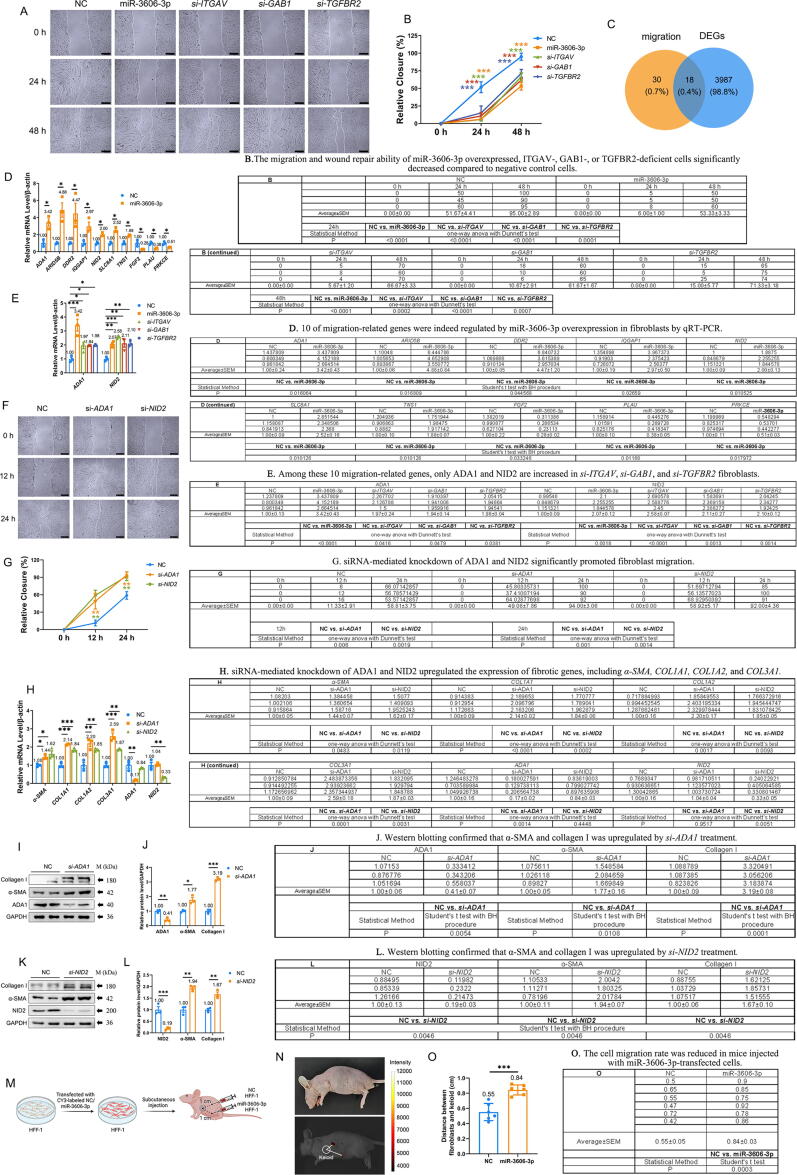
**(Relative to Stage 3). MiR-3606-3p overexpression, ITGAV, GAB1, and TGFBR2 knockdown repress fibroblast migration.** (**A**) Cell migration assays were evaluated by morphology and (**B**) relative closure distance of cell migration in primary fibroblasts with miR-3606-3p overexpression, ITGAV, GAB1, or TGFBR2 knockdown. Scale bar, 500 μm. (**C**) Venn diagram analysis of the overlap between DEGs and fibroblast migration genes. (**D**) Upregulated and downregulated fibroblast migration genes in miR-3606-3p-overexpressing fibroblasts compared with NC. (**E**) Elevated levels of *ADA1* and *NID2* in miR-3606-3p overexpression, ITGAV, GAB1, and TGFBR2 knockdown fibroblasts. (**F–G**) Cell migration assay results in primary fibroblasts with *si-ADA1* and *si-NID2*. Scale bar, 500 μm. (**H**) Increased mRNA levels of fibrotic genes after *si-ADA1* and *si-NID2* treatment. The experiment was repeated three independently times in D-H. (**I–J**) Protein levels of ADA1, α-SMA, and collagen I were detected by western blotting. (**K–L**) Protein levels of NID2, α-SMA, and collagen I were detected by western blotting. The experiments were repeated in triplicate in I-L. (**M−O**) Diagram and live imaging to assess the cell migration rate *in vivo*. N = 6. Data in B, E, G, and H were evaluated by one-way ANOVA (Dunnett’s test). Data in D, J, and L were evaluated using the Student's *t*-test and corrected using the Benjamini-Hochberg procedure. Data in O were evaluated using the Student's *t*-test. Data represented as mean ± SEM. *P < 0.05; **P < 0.01; ***P < 0.001.

When skin injury occurs, excessive fibroblast cells migrate to the loci, leading to uncontrolled ECM deposition [33], [34]. To investigate the inhibitory effect of miR-3606-3p on cell migration, we performed scratch assays on primary SSc fibroblasts. Overexpression of miR-3606-3p lowered the wound repair ability as evaluated by scratch distance measurement, which is consistent with the identification of the fibroblast migration pathway in the GO analysis in Fig. 2D. Similarly, the migration and wound repair ability of ITGAV-, GAB1-, or TGFBR2-deficient cells was significantly decreased compared to those of negative control cells (Fig. 6**A–B**). Collectively, these results suggested that miR-3606-3p may block ITGAV-, GAB1-, and TGFBR2-induced fibroblast migration.

To reveal the migration-related genes responsible for this inhibitory effect, we first performed a GO search for all fibroblast migration-related genes. Subsequently, we performed a Venn diagram analysis using the Venny 2.1 program for 48 fibroblast cell migration-related genes and 4,005 DEGs induced by miR-3606-3p. A total of 18 fibroblast cell migration-related genes were identified to be dysregulated in miR-3606-3p-transfected skin fibroblasts (Fig. 6**C**). Next, we performed qRT-PCR to verify that 10 of these genes were indeed regulated by miR-3606-3p overexpression in fibroblasts (Fig. 6**D**). Among these miR-3606-3p-regulated genes, adenosine deaminase (*ADA1*) and nidogen-2 (*NID2*), two known migration- and fibrosis-inhibitory factors [35], were unregulated 3.42- and 2.07-fold higher, respectively. Our results confirm that only *ADA1* and *NID2* are increased in *si-ITGAV*, *si-GAB1*, and *si-TGFBR2* fibroblasts, all nearly or more than two-fold increase (Fig. 6**E and** Supplemental Fig. S4**A**). Moreover, siRNA-mediated knockdown of *ADA1* and *NID2* significantly promoted fibroblast migration (Fig. 6**F–G**) and upregulated the expression of fibrotic genes, including *α-SMA*, *COL1A1*, *COL1A2*, *and COL3A1* (Fig. 6**H**). Western blotting further confirmed that α-SMA was upregulated 1.77- and 1.94-fold by *si-ADA1* and *si-NID*, respectively, and collagen I was upregulated 3.19- and 1.67-fold by *si-ADA1* and *si-NID*, respectively (Fig. 6**I–L**).

To further verify the effect of miR-3606-3p in inhibiting cell migration *in vivo*, we constructed a keloid-bearing mouse model by injecting CY3-transfected HFF-1 cells (Fig. 6**M**). The transfection efficiency of NC and miR-3606-3p were equivalent, as shown in Supplemental Fig. S4**B**. Live animal imaging revealed a 1.53-fold increase in the distance between fibroblasts and keloid (P = 0.0003), suggesting that the cell migration rate was reduced in mice injected with miR-3606-3p-transfected cells (Fig. 6**N–O**). Collectively, these results confirm that miR-3606-3p inhibits cell migration and suggest that ITGAV, GAB1, and TGFBR2 promote cell migration by regulating the expression of fibroblast cell migration-associated genes.

### MiR-3606-3p arrested cell cycle further inhibited cell proliferation by mainly targeting TGFBR2

**Fig. 7:**
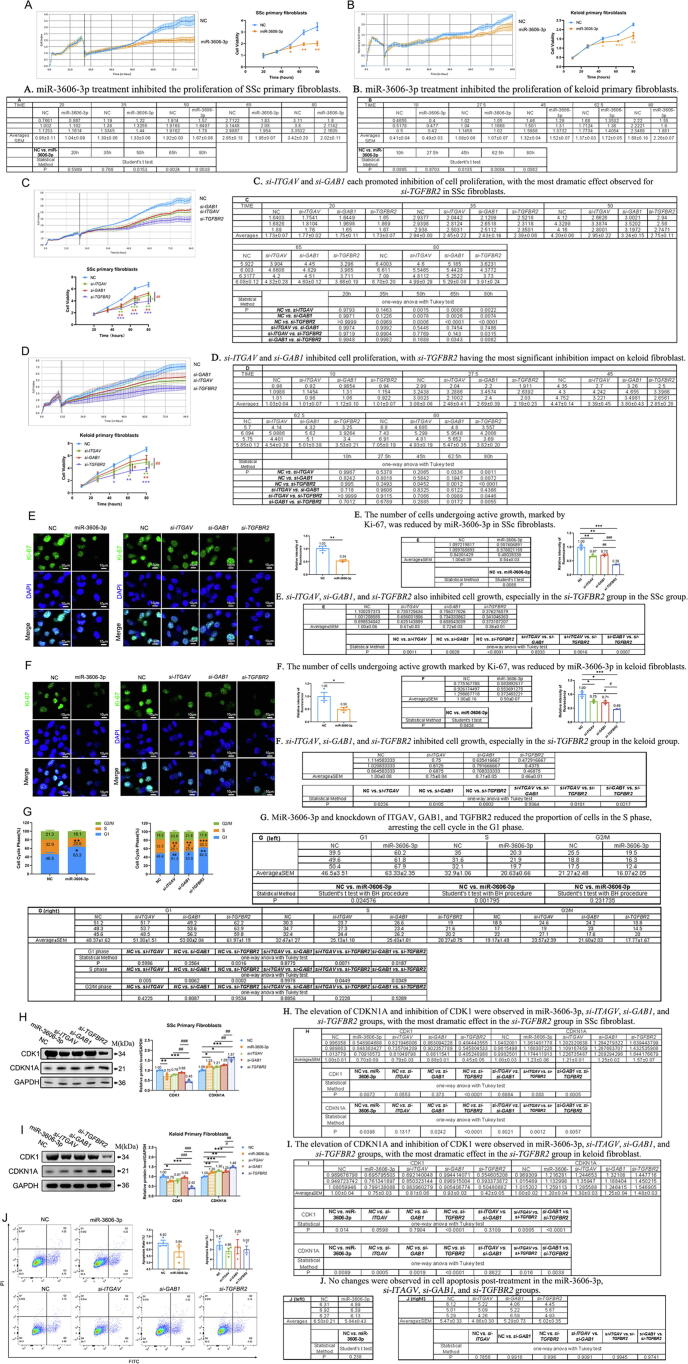
**(Relative to Stage 3). TGFBR2 knockdown and miR-3606-3p inhibit fibroblast proliferation by inhibiting the cell cycle.** (**A–B**) RTCA assay of the cell proliferation ability between NC and miR-3606-3p overexpression groups in SSc and keloid primary fibroblasts. (**C–D**) Cell proliferation ability assessed by RTCA assay in *ITGAV, GAB1*, and *TGFBR2* knockdown SSc and keloid primary fibroblasts. (**E–F**) Immunocytochemistry of Ki-67 in NC, miR-3606-3p-overexpressed, *si-ITGAV*, *si-GAB1*, and *si-TGFBR2* SSc and keloid primary fibroblasts. (**G**) Quantification of the cell cycle phase after primary fibroblasts were treated with miR-3606-3p-overexpression, *si-ITGAV*, *si-GAB1*, or *si-TGFBR2* for 72 h. The experiments were performed in triplicate in A-G. (**H–I**) The CDK1 and CDKN1A expression in SSc and keloid primary fibroblasts. The experiments were performed in triplicate. (**J**) Flow cytometry bivariate density plots and quantification of apoptosis after primary fibroblasts were treated with miR-3606-3p-overexpression, *si-ITGAV*, *si-GAB1*, and *si-TGFBR2* for 72 h. The experiments were performed in triplicate. Scale bar, 10  μm. Data in A, B, E (upper panel), F (upper panel), G (upper panel), and J (left panel) were evaluated by Student's *t*-test. Data in C, D, E (lower panel), F (lower panel), G (lower panel), H, I, and J (right panel) were evaluated by one-way ANOVA (Tukey’s test). Data represented as mean ± SEM. *P < 0.05; **P < 0.01; ***P < 0.001 versus control. # P < 0.05; ##P < 0.01; ###P < 0.001 versus different treatment groups.

The abnormal proliferation of fibroblasts is a key step in the development of skin fibrosis. Our RNA-seq results indicated that the overexpression of miR-3606-3p significantly inhibits the mitotic cell cycle pathway in fibroblasts (Fig. 2D). Therefore, we speculated that miR-3606-3p may regulate cell proliferation by targeting ITGAV, GAB1, and/or TGFBR2. RTCA confirmed the inhibitory effects of miR-3606-3p on the proliferation of SSc and keloid primary fibroblasts (Fig. 7**A–B**). Furthermore, *si-ITGAV* and *si-GAB1* each promoted modest inhibition of cell proliferation, with the most dramatic effect observed for *si-TGFBR2* (Fig. 7**C–D**). Similarly, in SSc and keloid primary fibroblasts, the number of cells undergoing active growth was reduced by 46 % and 50 %, respectively, by miR-3606-3p as assessed by Ki-67. In addition, *si-ITGAV*, *si-GAB1*, and *si-TGFBR2* also inhibited cell growth, especially in the *si-TGFBR2* group (SSc fibroblasts: 33 %, 28 %, and 64 % decrease, respectively; keloid fibroblasts: 25 %, 29 %, and 54 % decrease, respectively) (Fig. 7**E–F**). For additional insight, we examined changes in the cell cycle by flow cytometry, which revealed that miR-3606-3p and knockdown of ITGAV, GAB1, and TGFBR2 reduced the proportion of cells in the S phase (32.9 % in NC *vs.* 20.6 % in miR-3606-3p; 32.5 % in NC *vs.* 25.1 % in *si-ITGAV*, 25.4 % in *si-GAB1*, and 20.3 % in *si-TGFBR2*), arresting the cell cycle in the G1 phase (46.5 % in NC *vs.* 63.3 % in miR-3606-3p; 48.4 % in NC *vs.* 51.3 % in *si-ITGAV*, 53.0 % in *si-GAB1*, and 62.0 % in *si-TGFBR2*) (Fig. 7**G** and Supplemental Fig. S5**A**).

Cyclin-dependent kinase inhibitor 1a (CDKN1A, P21) is an important factor in G1 phase arrest that inhibits the essential mammalian cell cycle protein CDK1 [36]. In SSc and keloid primary fibroblasts, abnormal elevation of CDKN1A and inhibition of CDK1 were observed in all groups, with the most dramatic effect in the *si-TGFBR2* group (Fig. 7**H–I** and Supplemental Fig. S5**B-C**). Additionally, there were no changes observed in cell apoptosis post-treatment (Fig. 7**J** and Supplemental Fig. S5**D–E),** suggesting that the effect of miR-3606-3p and *si-TGFBR2* are specific for the cell cycle. The above results indicate that miR-3606-3p may inhibit cell cycle progression and fibroblast proliferation primarily by targeting TGFBR2.

### MiR-3606-3p overexpression improves skin fibrosis *in vivo*

**Fig. 8:**
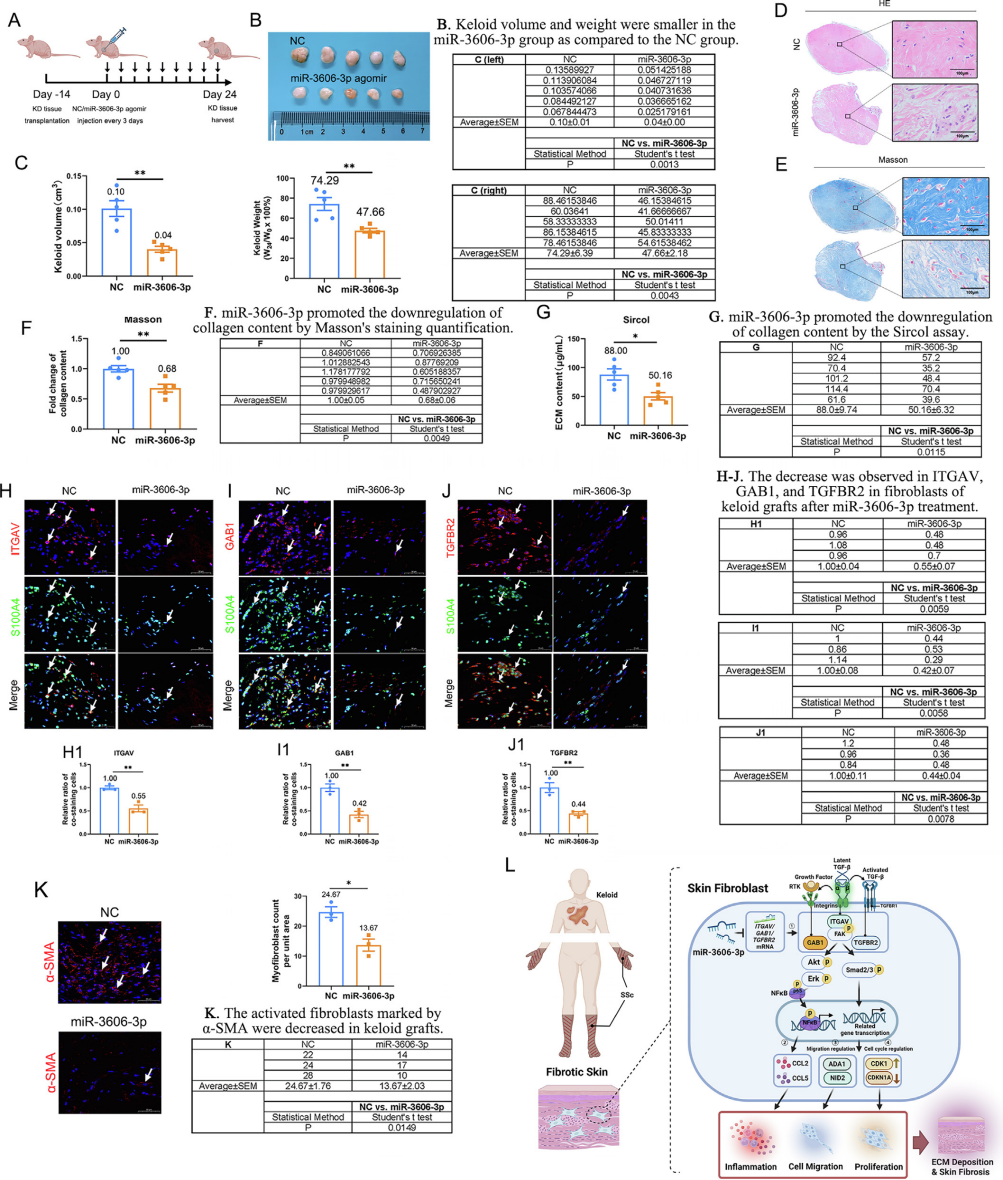
**(Relative to Stage 4). Overexpression of miR-3606-3p alleviates skin fibrosis *in vivo*.** (**A**) The construction of a keloid-bearing mouse model. (**B**) Morphological evaluation and (**C**) quantitative analysis of subcutaneous keloid grafts (5 NC *vs.* 5 miR-3606-3p). (**D–E**) H&E and Masson’s staining of keloid grafts. Scale bar, 100 μm. (**F**) Quantitative analysis of collagen content in Masson’s staining. (**G**) ECM content in NC and miR-3606-3p grafts by sircol assay. (**H–J**) Dual immunofluorescence staining and (**H1–J1**) quantification of ITGAV, GAB1, TGFBR2, and S100A4 in keloid grafts after NC or miR-3606-3p treatment. The experiments were performed in triplicate. Scale bar, 50  μm. (**K**) Immunofluorescence staining and quantification of α-SMA in keloid grafts after NC or miR-3606-3p treatment. Scale bar, 50 μm. N = 3 per group. Student's *t*-test analyzed all comparisons. Data represented as mean ± SEM. *P < 0.05; **P < 0.01; ***P < 0.001. (**L**) Model of the mechanism of miR-3606-3p in inducing anti-inflammatory and antifibrotic effects via the integrin, TGF-β, and ERK/AKT pathways.

Given our observed ability of miR-3606-3p to inhibit collagen deposition, inflammation, cell migration, and proliferation in dermal fibroblasts, we investigated the possibility that miR-3606-3p upregulation may provide an approach to alleviate skin fibrosis *in vivo*. To this end, we injected keloid-bearing mice with NC or miR-3606-3p every three days and harvested grafts on day 24 (Fig. 8**A**). Subcutaneous keloid grafts were morphologically smaller in volume (FC =  − 2.5-fold, P = 0.0013) and weight (FC =  − 1.6-fold, P = 0.0043) in the miR-3606-3p group as compared to the NC group (Fig. 8**B–C**). Histologically, miR-3606-3p treatment reduced the density of dermal tissue (Fig. 8**D**). Furthermore, Masson's staining and Sircol assays showed that miR-3606-3p promoted the release of collagen fiber bundles and the downregulation of collagen content (FC =  − 1.8-fold, P = 0.0115) (Fig. 8**E-G**). Immunofluorescence staining assays also demonstrated a nearly 50 % decrease in ITGAV, GAB1, and TGFBR2 in keloid fibroblasts after miR-3606-3p treatment (Fig. 8**H-J**), which is consistent with *in vitro* experiments. Moreover, miR-3606-3p treated keloid showed a decrease in α-SMA-positive myofibroblasts (Fig. 8**K**). To further confirm the protective role of miR-3606-3p in skin fibrosis, we downregulated miR-3606-3p using a specific inhibitor and found that the inhibitor significantly promoted fibrogenesis, inflammation, migration, and proliferation and aggravated skin fibrosis in a humanized mouse transplanted keloid model (Supplemental Fig. S6**A–I**). Collectively, these results demonstrate that miR-3606-3p diminishes ITGAV, GAB1, and TGFBR2 expression and alleviates skin fibrosis *in vivo*. Here, we summarized the anti-fibrosis mechanism of miR-3606-3p in skin fibrosis in Fig. 8**L**. ① miR-3606-3p targets the 3′-UTRs of *ITGAV*, *GAB1*, and *TGFBR2* mRNA and subsequently inhibits their protein expression, which attenuates the integrin-mediated phosphorylation of FAK, AKT, ERK1/2, and SMAD2/3. ② p-AKT/ERK1/2 triggers inflammatory responses by activating the NF-κB signaling pathway, leading to the release of inflammatory factors, including CCL2 and CCL5. ③ Two important negative regulators of fibroblast migration, ADA1 and NID2, are inhibited by p-AKT/ERK1/2/SMAD2/3, resulting in collagen deposition and cell migration. ④ Cell cycle arrest is mediated by the regulation of CDK1 and CDKN1A. ⑤ miR-3606-3p inhibition of ITGAV, GAB1, and TGFBR2 expression eventually leads to attenuation of collagen deposition, cell proliferation, migration, and inflammation.

## Discussion

Treatment options for skin fibrosis are limited. The present study identifies a previously unrecognized role of miR-3606-3p in fibroblast abnormalities that lead to skin fibrosis. First, a decrease in miR-3606-3p was observed in skin tissue and primary fibroblasts from patients with skin fibrosis, including both systemic sclerosis and keloids, and this decrease was correlated with the severity of the disease. Second, miR-3606-3p was shown to inhibit a wide range of pathological phenotypes of fibroblasts, including excessive proliferation, inflammation, migration, activation, and collagen deposition. Third, miR-3606-3p was demonstrated to inhibit the integrin/FAK, p-ERK/p-AKT, and p-SMAD2/3 pathways by directly targeting the 3′-UTRs of GAB1, ITGAV, and TGFBR2. Fourth, ITGAV overexpression activated integrin/FAK, which further induced GAB1-dependent p-AKT/p-ERK to trigger inflammatory responses and TGFBR2-dependent p-SMAD2/3. Finally, miR-3606-3p alleviated skin fibrosis in a humanized mouse model of transplanted keloid. Overall, our results suggest that miR-3606-3p inhibits integrin-mediated activation of integrin/p-FAK, p-AKT/p-ERK, and TGF-β signaling by targeting ITGAV, GAB1, and TGFBR2 to inhibit fibrogenesis, inflammation, migration, and proliferation of fibroblasts and alleviate skin fibrosis (Fig. 8L).

Accumulated evidence indicates that miRNAs are involved in skin fibrosis. However, these studies are mostly limited to regulating fibroblast monofunctionality. The abnormal phenotypes of fibroblasts vary in different stages of skin fibrosis, such as inflammation in the early stages, migration and proliferation in intermediate and late stages, and high collagen deposition in late stages [37], [38]. Existing studies lack a comprehensive understanding of the role of miRNAs in multi-stage cell phenotypic abnormalities throughout the disease process, which severely limits the clinical application of miRNAs. In this study, we observed the downregulation of miR-3606-3p in both SSc and keloid dermal tissues, which negatively correlated with the MRSS score, suggesting that miR-3606-3p plays a crucial part in various skin fibrotic diseases. Moreover, we discover that miR-3606-3p exerts a potent antifibrotic impact by inhibiting collagen deposition, chemokine and cytokine synthesis, fibroblast migration, and proliferation in both SSc and keloids through the regulation of three novel targets. These findings suggest that miR-3606-3p may function as a regulator of multidimensional abnormalities in fibrosis.

Previous studies have suggested that hyperactivation of integrin, AKT/ERK1/2, and TGF-β signaling is involved in skin fibrosis. However, the underlying mechanisms are not fully understood. We directly demonstrated that miR-3606-3p decreases *ITGAV, GAB1*, and *TGFBR2* expression and enriches focal adhesion, PI3K/AKT, ERK1/2, and TGF-β pathways. Furthermore, we identified some notable differences in the ITGAV, GAB1, and TGFBR2 signaling mechanisms, with upregulation of ITGAV and GAB1 primarily resulting in fibroblast inflammation and upregulation of TGFBR2 primarily inducing fibroblast proliferation. Our results are supported by previous studies demonstrating that ITGAV, GAB1, and TGFBR2 contribute to skin fibrosis [14], [21], [39], [40], [41]. More importantly, we elucidated the common and distinct different effects of miR-3606-3p, ITGAV, GAB1, and TGFBR2 in regulating fibroblast abnormalities. Mechanistically, we clarified the crosstalk between the ITGAV-, GAB1- and TGFBR2-mediated integrin/FAK, p-ERK/p-AKT, and p-SMAD2/3 pathways, starting from the upstream integrin pathway and proceeding towards the downstream p-AKT/p-ERK and p-SMAD2/3 pathways. Our findings are consistent with previous studies demonstrating that ITGAV is an important integrin protein that triggers RTK and TGF-β signaling to activate fibroblasts by interacting with the RTK signaling ligand or receptor [25], [26], [42], [43]. These published results also confirm the rationality of our discovery that these signals exist in crosstalk. More interestingly, we also demonstrate the essential role of GAB1 and TGFBR2 in the ITGAV/integrin-induced AKT/ERK1/2 and TGF-β/SMAD2/3 pathways.

The next critical question is how the disrupted miR-3606-3p/ITGAV/GAB1/TGFBR2 axis induces fibroblast abnormalities and further contributes to skin fibrosis. It has been well established that fibroblast inflammation and migration each contribute to the collagen deposition of fibroblasts. The cytokines produced by inflammatory cells activate proinflammatory signals in fibroblasts, further evoking profibrotic pathways and eventually promoting collagen synthesis [9], [44]. For example, IL-33 induces the polarization of naive T cells to Th2 cells, which consequently releases IL-31 [45]. In response, IL-31 induces the production of proinflammatory cytokines CCL2 and CCL5 in fibroblasts, which further triggers the TGF-β, CTGF, and PDGF pathways [46]. Moreover, the role of the IL-33/IL-31 axis in various immune-mediated diseases, including SSc, has been confirmed [47]. Besides inflammation, migration, and proliferation also play important roles in fibroblast collagen synthesis. In response to injury stimuli, peripheral fibroblasts migrate to repair impaired tissue, and profibrotic pathways are activated, releasing ECM [33]. Cell proliferation behavior directly provides more ECM-producing cells, strongly promoting fibrosis progression [48]. Therefore, fibroblast migration, inflammation, and cell growth are all important therapeutic targets for reducing collagen deposition and alleviating skin fibrosis. Notably, we demonstrated that miR-3606-3p targets each of these processes.

In active keloids, pro-migration factors, such as TGF-β, PDGF, and TNF-α, can be released to trigger fibroblast migration [49]. Methodologically, the study of cell migration is often limited to *in vitro* experiments. Therefore, we developed a strategy to monitor the *in vivo* fibroblast migration rate based on *in vivo* imaging technology in mice. Due to the absence of a homolog of miR-3606-3p in mice, we constructed a humanized keloid-bearing mouse model in which the distance between the fluorescent edge of CY3 and the keloid reflects the rate of cell migration, subtly reflecting the inhibitory effect of miR-3606-3p on cell migration *in vivo*. MiR-3606-3p overexpression, ITGAV, GAB1, and TGFBR2 knockdown inhibited cell migration by promoting the expression of *ADA1* and *NID2*. Interestingly, increased collagens were found in both *si-ADA1-* and *si-NID2-*mediated migratory fibroblasts. Additionally, the cell proliferation behavior was inhibited by overexpression of miR-3606-3p and its downstream targets. We demonstrated that miR-3606-3p-TGFBR2-SMAD2/3 signaling inhibits cell proliferation by blocking the cell cycle rather than affecting apoptosis. Therefore, the effects of miR-3606-3p are comprehensive yet specific.

Another thrilling finding in this report is the investigation into the therapeutic potential of miR-3606-3p in treating skin fibrosis. We confirmed that overexpression of miR-3606-3p in the keloid-bearing mouse model improved skin fibrosis in terms of morphology and histology. In contrast, inhibiting miR-3606-3p aggravated skin fibrosis. Both our in vitro and in vivo results showed that miR-3606-3p alleviated fibroblast inflammation, fibrogenesis, cell migration, and proliferation by inhibiting ITGAV, GAB1, and TGFBR2 expression. Evidence has shown the combination of ERK1/2 and PI3K/AKT/mTOR inhibitors may be an effective strategy for the treatment of clinical drug resistance in cancer [50], and clinical trials of TGF-β pathway inhibitors for tumor therapy are also being conducted [51]. However, the use of these inhibitors is limited due to their high toxicity and the poor specificity of the targeted catalytic domain, and kinase targeting often results in drug resistance [52]. Additionally, it would theoretically be beneficial for alleviating skin fibrosis to simultaneously target multiple pathways, including integrin/ERK/AKT/SMAD, and our results indicated that this strategy could be possible by targeting miR-3606-3p. The multi-target and multi-functional effect of miR-3606-3p theoretically expands the clinical applicability of miR-3606-3p. Patients with single or multiple high expression of ITGAV, GAB1, and TGFBR2 will all be indicated for miR-3606-3p therapy, helping to reduce the economic and organ toxicity burden of administering multiple drugs. Moreover, the miR-3606-3p overexpression strategy could also potentially be used to treat NF-κB-activated diseases, such as cancer and neurodegenerative diseases, and other types of organ fibrosis with FAK, AKT, ERK, and SMAD activation, like pulmonary and liver fibrosis [53], [54]. Future studies to evaluate the activity of miR-3606-3p expression in other diseases, including additional fibrosis models, will help to determine the extent of its therapeutic potential.

## An overview of this study

### The rationale and necessity of this study

Treatment options for skin fibrosis are limited, and fibroblast abnormalities play a pivotal role in the pathogenesis of skin fibrosis. A better understanding of the mechanisms underlying fibroblast abnormalities and developing antifibrotic strategies that aim to alleviate or revert the disease process is needed. Our earlier research has shown that miR-3606-3p inhibits collagen deposition and fibroblast proliferation, exhibiting an antifibrotic impact in systemic sclerosis. In fact, in order to encourage skin fibrosis, fibroblasts also mediate migration, differentiation, inflammation, immunology, and other processes. These findings motivated us to look into the processes and target genes of miR-3606-3p in skin fibrosis to understand its biological function better, including its potential use as a therapeutic target and biomarker.

### The novelty and significance of this study

We clarified a compressive fibroblast abnormality in skin fibrosis, including excessive collagen deposition, enhanced cell migration, persistent inflammation, and uncontrolled proliferation. We next discovered the essential role of miR-3606-3p in regulating these fibroblast abnormalities through its multi-targeting capabilities. Mechanistically, miR-3606-3p attenuates integrin/FAK, AKT, ERK, and SMAD pathways by directly targeting ITGAV, GAB1, and TGFBR2. ITGAV/FAK-activated GAB1/AKT/ERK and TGFBR2/SMAD2/3 pathways induced inflammation and proliferation, respectively, but had a common effect on promoting fibroblast fibrogenesis and migration. These findings indicated that miR-3606-3p may serve as a novel target for simultaneously affecting multiple pathways that lead to diverse fibrotic diseases. In addition, we developed a mouse imaging model for detecting fibroblast migration *in vivo* and a nude mouse model for assessing skin fibrosis under scar stress.

### The major experimental stages

Our experiments and discoveries can be divided into four stages:

Stage 1. We observed a decrease of miR-3606-3p in skin fibrosis, which showed a negative correlation with disease severity (Relative to Fig. 1).

Stage 2. Our results showed that miR-3606-3p targets the 3′-UTRs of ITGAV, GAB1, and TGFBR2 in fibroblasts (Relative to Fig. 2, Fig. 3).

Stage 3. We demonstrated that miR-3606-3p downregulation activates ITGAV/FAK, GAB1/AKT/ERK, and TGFBR2/SMAD2/3. ITGAV/FAK and GAB1/AKT/ERK induce fibroblast fibrogenesis, migration, and inflammation. In contrast, ITGAV/FAK and its activated TGFBR2/SMAD2/3 signaling pathway induce fibroblast fibrogenesis, migration, and proliferation (Relative to Fig. 4, Fig. 5, Fig. 6, Fig. 7).

Stage 4. We employed a keloid mouse model to demonstrate that miR-3606-3p effectively alleviates skin fibrosis *in vivo* (Relative to Fig. 8)“.

## Conclusions

In the first stage of this study, we revealed that fibroblasts undergo a wide range of abnormalities in skin fibrosis, including fibrogenesis, migration, inflammation, and proliferation. We went on to find that miR-3606-3p was significantly downregulated in these abnormal fibroblasts (P = 0.0278, 92.2 % decrease in SSc fibroblasts, and P = 0.0021, 52.2 % decrease in keloid fibroblasts) and negatively correlated with disease severity (The correlation coefficient between miR-3606-3p and MRSS was *−* 0.71, P = 0.0002.). In conclusion, we report a never-before-reported microRNA, miR-3606-3p, which is lowly expressed in skin fibrosis and may play a role in disease severity monitoring.

In the second experimental stage of this study, we demonstrated that miR-3606-3p was able to target binding to *ITGAV*, *GAB1*, and *TGFBR2* via their 3′-UTRs, thereby decreasing their expression (74 %, 75 %, and 69 % decrease at mRNA levels, respectively. p = 0.0004 for all.). We are the first to discover that ITGAV, GAB1, and TGFBR2 can be simultaneously regulated by miR-3606-3p.

In the third experimental stage, to elucidate the role of miR-3606-3p in regulating fibroblast abnormalities, we further found that miR-3606-3p could simultaneously inhibit fibroblast excessive fibrogenesis (28.4 % decrease of ECM content in SSc, P = 0.006; 46.9 % decrease of ECM content in keloid, P = 0.0086), inflammation (27.5 % decrease of p-p65 level in SSc, P < 0.001), migration (88.4 % decrease in SSc, P < 0.0001), and proliferation (40.9 % decrease in SSc, P = 0.0038; 25.7 % decrease in keloid, P = 0.0062.), suggesting an essential role for miR-3606-3p in regulating fibroblast functional homeostasis. Mechanistic studies revealed that miR-3606-3p could simultaneously inhibit the ITGAV-mediated integrin/p-FAK pathway, the GAB1-mediated p-AKT/p-ERK pathway, and the TGFBR2-mediated TGF-β/p-SMAD2/3 pathway. We found for the first time that miR-3606-3p can regulate three signaling pathways simultaneously through the above three target genes and regulate different abnormal fibroblast behaviors through different signaling pathways. These fibroblast abnormalities that miR-3606-3p regulated are early, late, or full-course mediators of skin fibrosis. Notably, unlike other microRNAs, the miR-3606-3p we identified could regulate all of these key features of SSc fibroblasts, making it reasonable that a miR-3606-3p therapy strategy may be suitable for all stages of skin fibrosis.

In the final experimental stage, we evaluated the potential clinical value of miR-3606-3p as a treatment for skin fibrosis. We employed a keloid mouse model and demonstrated that miR-3606-3p overexpression effectively alleviated skin fibrosis in keloid-bearing nude mice (60.0 % decrease in keloid volume, P = 0.0013). In contrast, miR-3606-3p inhibition significantly exacerbated skin fibrosis in keloid-bearing nude mice (46.7 % increase in keloid volume, P = 0.0001). The results of this stage suggest that developing a miR-3606-3p intervention strategy may aid in diagnosing and treating skin fibrosis.

In summary, our research has achieved our proposed goal and the experimental objectives, which were devoted to evaluating the roles and mechanisms of miR-3606-3p in regulating multiple fibroblast abnormalities and its therapeutic potential in skin fibrosis”.

## Declaration of competing interest

The authors declare that they have no known competing financial interests or personal relationships that could have appeared to influence the work reported in this paper.
