# Supplementary material for: miR-3606-3p alleviates skin fibrosis by integratively suppressing the integrin/FAK, p-AKT/p-ERK, and TGF-β signaling cascades
Source: J Adv Res. 2024 Nov 20;75:271–90. doi: 10.1016/j.jare.2024.11.027 (PMC12536609; doi:10.1016/j.jare.2024.11.027)
Supplement: Supplementary Data 1 [file mmc1.docx]

**Supplemental Figures and Tables**

**Supplemental Figures**

**Supplemental Figure S1**


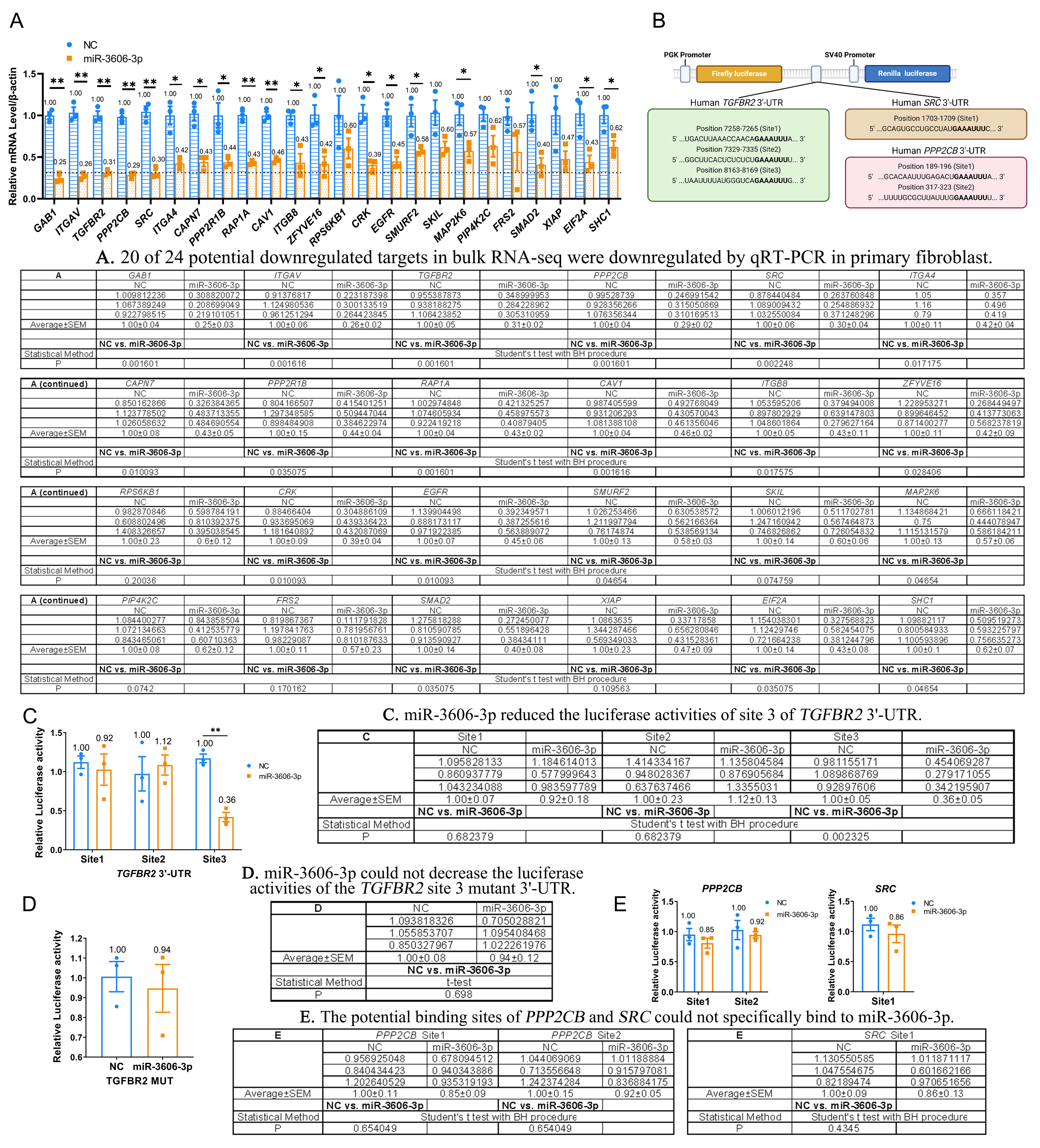


**Supplemental Figure S1. Identifying miR-3606-3p target genes** (**A**) The mRNA levels of genes annotated in the integrin, TGFBR, PI3K/AKT, and ERK1/2 pathways. (**B**) Potential miR-3606-3p binding sites in TGFBR23'-UTR. (**C**) Luciferase assay of TGFBR2 potential binding sites in primary fibroblasts. (**D**) Luciferase assay of site 3 mutation of TGFBR2 3'-UTR. (**E**) Luciferase assay of other potential target genes in primary fibroblasts. The experiments in A-E were performed in triplicate. Data represented as median±SEM. All comparisons were analyzed using the Student’s t-test. P values in A, C, and E (left panel) were corrected using the Benjamini-Hochberg procedure. *P < 0.05; **P < 0.01; ***P < 0.001.

**Supplemental Figure S2**


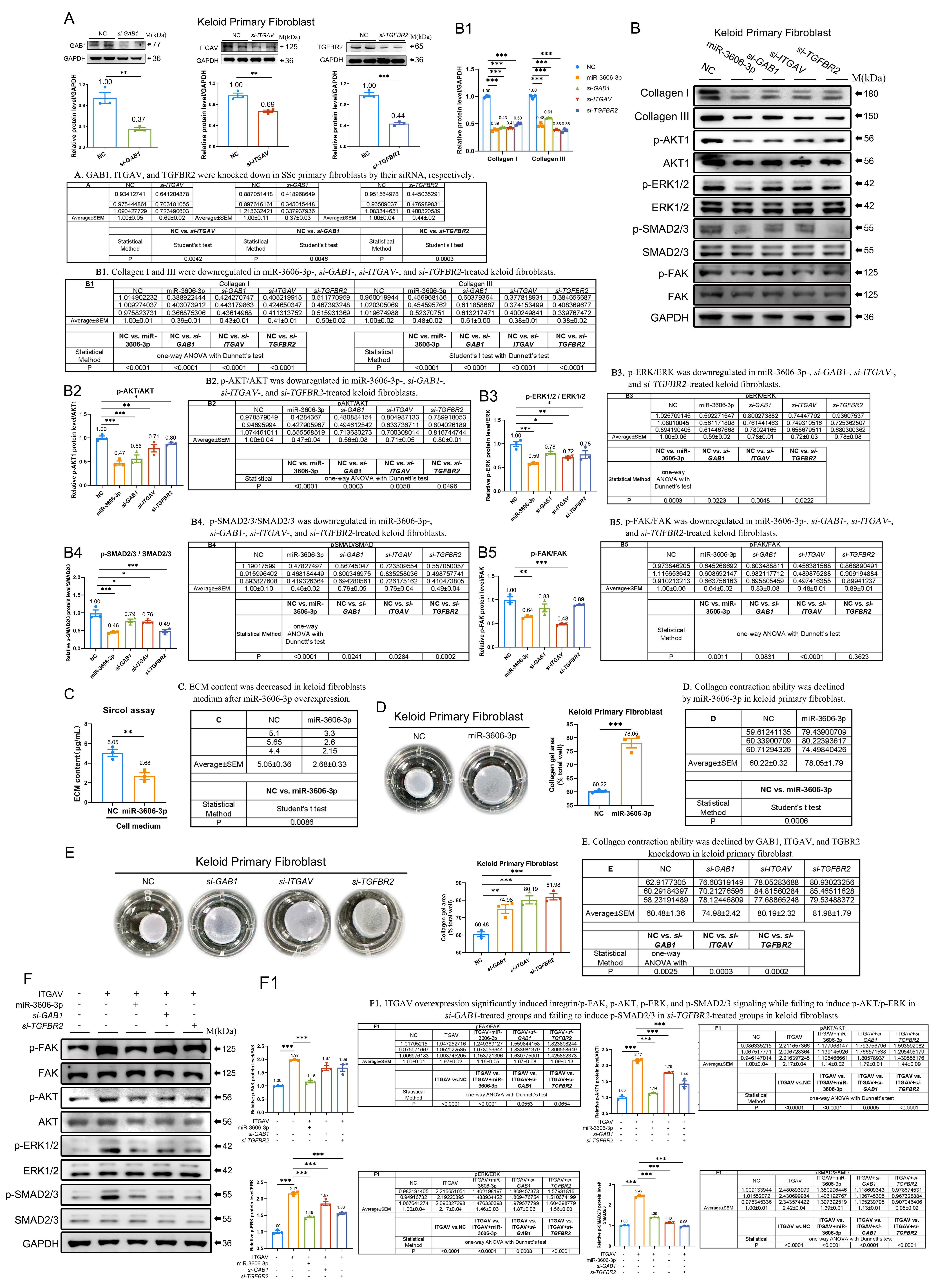


**Supplemental Figure S2. The activation inhibition of the GAB1-mediated ERK/AKT pathway and the TGFBR2-mediated SMAD2/3 pathway by miR-3606-3p targeting ITGAV suppressed collagen synthesis.** (**A**) Western blot analysis of si-GAB1, si-ITGAV, and si-TGFBR2 in keloid primary fibroblasts. (**B**) Detection and (**B1-B5**) quantification of type I collagen, type III collagen, p-AKT, AKT, p-ERK1/2, ERK1/2, p-SMAD2/3, SMAD2/3, p-FAK and FAK levels in keloid primary fibroblast. (**C**) Sircol assay in keloid fibroblast medium with NC and miR-3606-3p. (**D-E**) Collagen contraction was detected in keloid fibroblasts transfected with miR-3606-3p, si-GAB1, si-ITGAV, or si-TGFBR2. (**F**) Western blot analysis and quantification of miR-3606-3p, si-GAB1, and si-TGFBR2 with ITGAV-overexpression in keloid primary fibroblasts. The experiments were performed in triplicate in A-F. Data represented as median±SEM. Student’s t-test evaluated data in A, C, and D. Data in B1-B5, E, and F1 were evaluated by one-way ANOVA (Dunnett’s test). *P < 0.05; **P < 0.01; ***P < 0.001.

**Supplemental Figure S3**

**
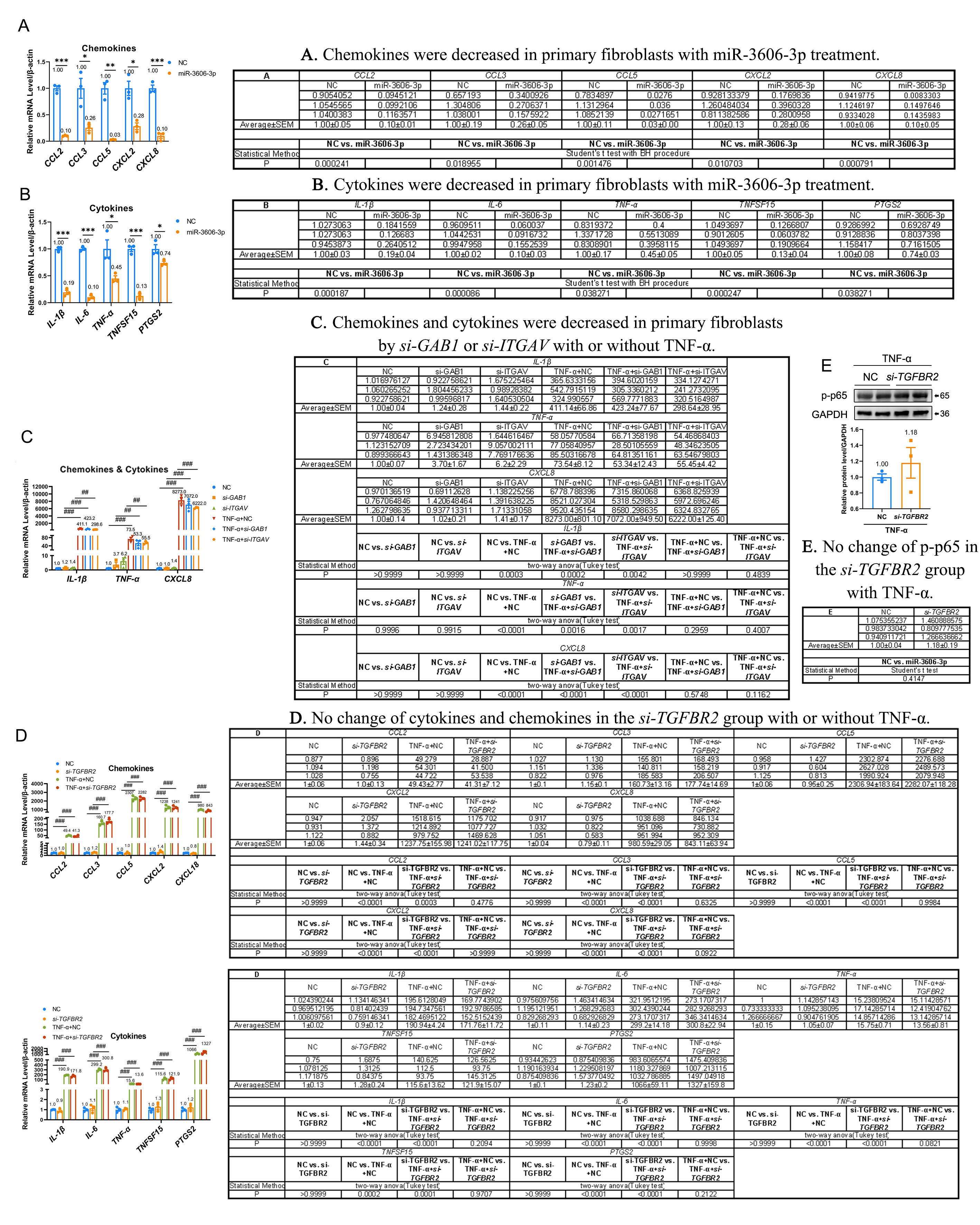
**

**Supplemental Figure S3. Detection of chemokine and cytokine levels with miR-3606-3p, *si-GAB1*, *si-ITGAV*, and *si-TGFBR2*.** (**A-B**) The decrease of chemokines and cytokines by miR-3606-3p overexpression. (**C**) The expression of cytokines after knockdown of *GAB1* and *ITGAV* with or without TNF-α. (**D**) The expression of chemokines and cytokines by *TGFBR2* knockdown with or without TNF-α treatment. (**E**) The expression of p-p65 in NC and TGFBR2 knockdown groups with TNF-α stimulation. The experiments were performed in triplicate in A-E. Data represented as median±SEM. Data in A, B, and E were evaluated using the Student’s t-test. P values in A and B were corrected using the Benjamini-Hochberg procedure. Data in C and D were evaluated by two-way ANOVA (Tukey’s test). *P < 0.05, **P < 0.01, ***P < 0.001 within different treatment groups. #P < 0.05, ##P < 0.01, ###P < 0.001 within *vs.* without TNF-α stimulation.

**Supplemental Figure S4**

**
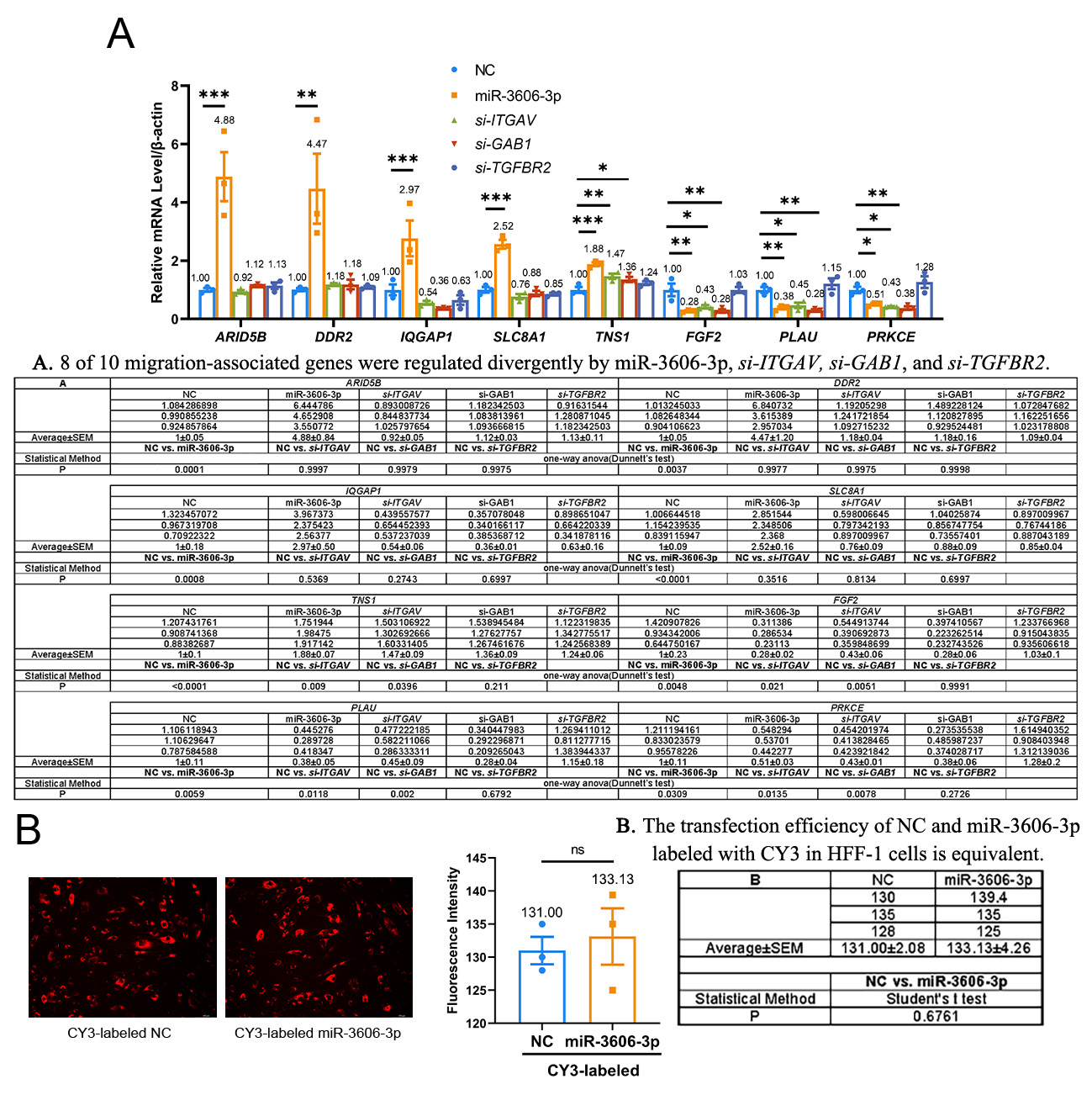
**

**Supplemental Figure S4. The expression of migration-related genes in SSc primary fibroblasts and the transfection efficiency of CY3-labeled NC and miR-3606-3p in HFF-1 cells.** (**A**) The expression of migration-related genes in miR-3606-3p overexpression, *si-ITGAV*, *si-GAB1*, and *si-TGFBR2* primary fibroblasts. (**B**) HFF-1 transfected with CY3-labeled NC and miR-3606-3p was observed by fluorescence microscopy to calculate the efficiency of transfection. The experiments were performed in triplicate in A-B. Data represented as median±SEM. Data in A were evaluated by one-way ANOVA (Dunnett’s test). Student’s t-test evaluated data in B. *P < 0.05; **P < 0.01; ***P < 0.001.

**Supplemental Figure S5**


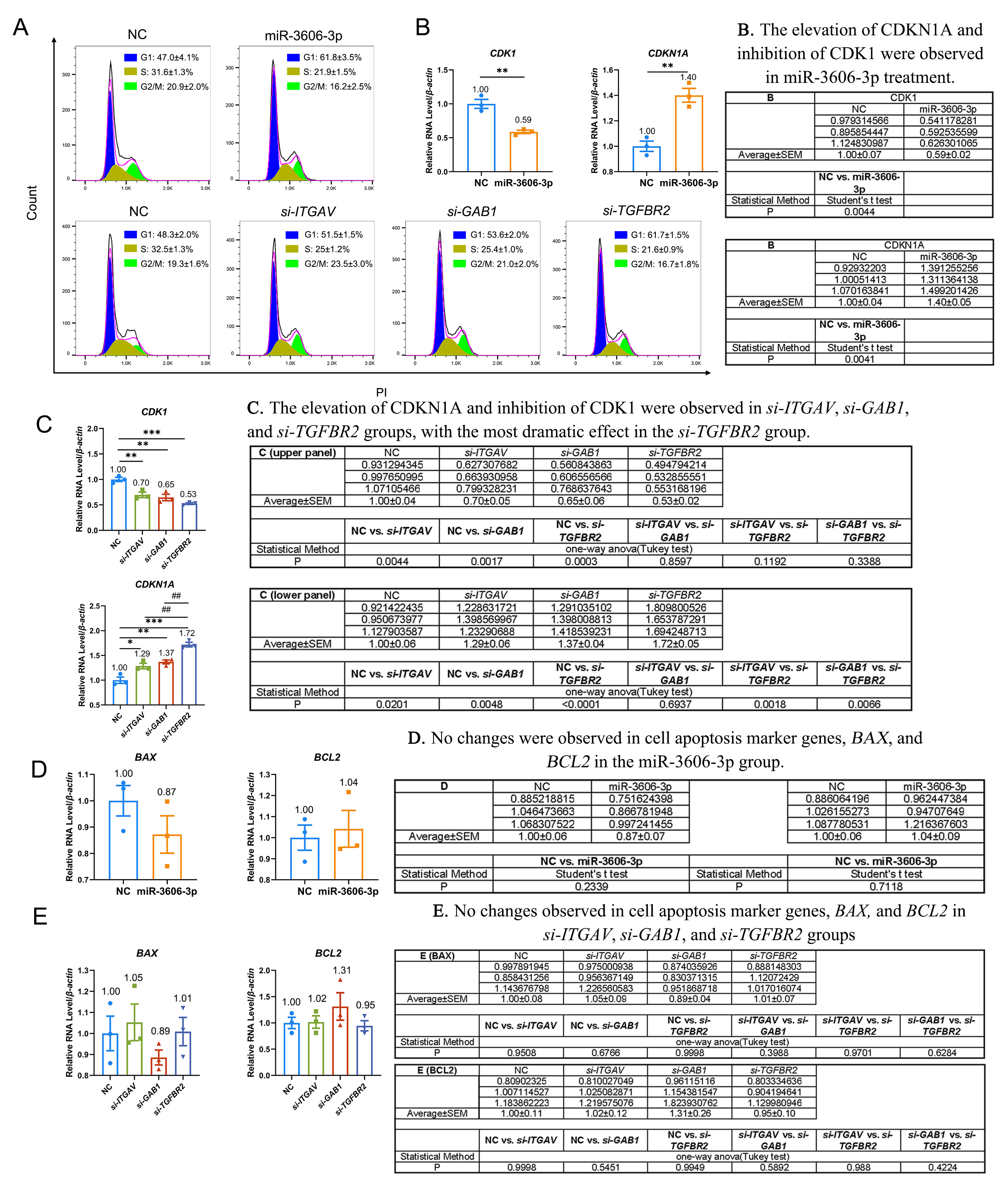


**Supplemental Figure S5. Cell proliferation detection in SSc and keloid primary fibroblast.** (**A**) Flow cytometric analysis, (**B-C**) cell cycle, and (**D-E**) apoptosis factor detection in miR-3606-3p overexpression, *ITGAV*, *GAB1*, and *TGFBR2* knockdown primary fibroblasts. The experiments were performed in triplicate in A-E. Data represented as median±SEM. Students' t-tests evaluated data in B and D. Data in C and E were evaluated by one-way ANOVA (Tukey’s test). *P < 0.05; **P < 0.01; ***P < 0.001 versus control. # P < 0.05; ##P < 0.01; ###P < 0.001 versus *si-TGFBR2* group.

**Supplemental Figure S6**


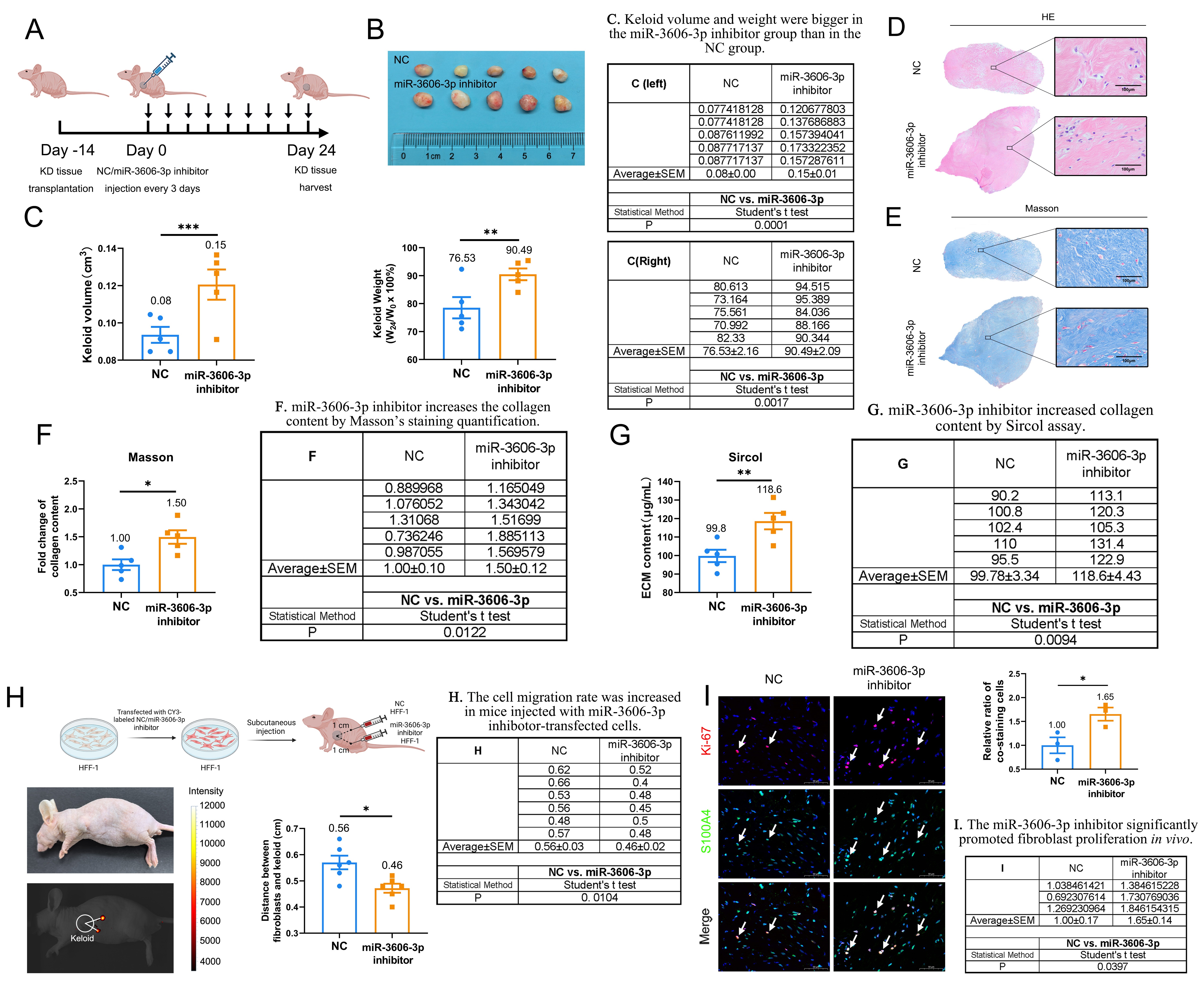


**Supplemental Figure S6. miR-3606-3p inhibition aggravates skin fibrosis *in vivo*.** (A) The injected strategy of a keloid-bearing mouse model. (**B**) Morphological evaluation and (**C**) quantitative analysis of subcutaneous keloid grafts (5 NC *vs.* 5 miR-3606-3p inhibitor). (**D**) H&E and (**E**) Masson’s staining of keloid grafts. Scale bar, 100 μm. (**F-G**) The quantification of collagen and ECM content, respectively, by Masson’s staining and Sircol assay. (**H**) Diagram and live imaging to assess the cell migration rate *in vivo*. N = 6. (**I**) Dual immunofluorescence staining and quantification of Ki-67 and S100A4 in keloid grafts after NC or miR-3606-3p inhibitor treatment. The experiments were performed in triplicate. Scale bar, 50 μm. Data represented as median±SEM. All comparisons were analyzed by Student’s t test. *P < 0.05; **P < 0.01; ***P < 0.001.

**Supplemental Tables**

**Supplemental Table S1**

| **Characteristic of SSc patients** |  |
| --- | --- |
| **Age (years), mean ± SD** | 36 ± 15 |
| **Gender** |  |
| Female | N=14 |
| Male | N=8 |
| **Disease subset** |  |
| Limited SSc | N=7 |
| Diffuse SSc | N=15 |
| **Disease duration** |  |
| Limited SSc | 7.0 ± 2.2 |
| Diffuse SSc | 7.5 ± 1.8 |
| **Modified Rodnan skin score (mRSS), mean ± SD** | 26.3 ± 10.1 |
| **Laboratory findings** |  |
| Anti-topoisomerase | N=12 |
| Anti-centromere antibody | N=4 |
| Anti-U1RNP antibody | N=4 |
| Anti-RNA polymerase antibody III | N=2 |
| **Lung involvement** | N=15 |
| ILD | N=15 |
| PAH | N=0 |
| **Drug taking history** |  |
| Anti-fibrosis drug | N=20 |
| Oral glucocorticoid | N=18 |
| Immunosuppressants | N=15 |
| Biologics | N=3 |
|  |  |
| **Characteristic of keloid patients** |  |
| **Age (years), mean ± SD** | 37 ± 11 |
| **Gender** |  |
| Female | N=10 |
| Male | N=16 |
| **Disease duration** | 2.5 ± 0.5 |
| **Site of Specimen** |  |
| Chest | N=12 |
| Ear | N=7 |
| Hip | N=4 |
| Shoulder | N=2 |
| Neck | N=1 |
| **Lung involvement** | N=0 |
| **Drug taking history** |  |
| Anti-fibrosis drug | N=0 |
| Oral glucocorticoid | N=20 |
| Immunosuppressants | N=0 |
| Biologics | N=0 |
| **N, Number of patients** |  |

**Supplemental Table S2**

| Name | Sequence (5’-3’) |
| --- | --- |
| RT primers |  |
| miR-3606-3p | GTCGTATCCAGTGCAGGGTCCGAGGTATTCGCACTGGATACGACCTAAGTAG |
| U6 | CGAGCACAGAATCGCTTCACGAATTTGCGTGTCAT |
| qRT-PCR Primers |  |
| *miR-3606-3p* | F: GTCGTATCCAGTGCAGGGT;  R: CGGCGAAAATTTCTTTCACTAC |
| *U6* | F: CGAGCACAGAATCGCTTCA;  R: CTCGCTTCGGCAGCACATAT |
| *ITGAV* | F: GACAGTCCTGCCGAGTA;  R: CTGGGTGGTGTTTGCT |
| *GAB1* | F: GAGGAGGAGATGAATAAGTGGGT;  R: AGGAGGTAGAGTAGCAGAGGATGA |
| *TGFBR2* | F: GTAGCTCTGATGAGTGCAATGAC;  R: CAGATATGGCAACTCCCAGTG |
| *ACTA2* | F: CTGAACGAGAACCAAGTGCG;  R: ACGAACCTCTTGCACATTTGA |
| *COL1A1* | F: GTGGGCAACGACTCTGGAC;  R: GCTTCGACATCAGCATTCCTCA |
| *COL1A2* | F: GATCACCCGAATGGCTATGAAT;  R: GGGGTCACAGTTGTCAATGTT |
| *COL3A1* | F: CACAACACGCTGTTCGGCTA;  R: CGATCCTGCATCTGTAAATCGC |
| *ADA1* | F: ACGATTGTAGTTGGAGGCTTG;  R: ATGGCTTCTTCGCTGACATCA |
| *ARID5B* | F: AAGATGTGCCGTCCTCATTGT;  R: ATCACCCCGTGCTTTACTTTC |
| *DDR2* | F: CTGGCGCTCAGCCATACAG;  R: CGCACTTATACTGGTCAAATCCC |
| *IQGAP1* | F: CCACCAGTGAACAGGAAGCA;  R: TTCTTTGGGTCCACGGTTCC |
| *NID2* | F: ACTACTCCGACTCCACTGTGA;  R: GATGTTCTGGTGGATGCGGT |
| *SLC8A1* | F: CACAGAAGATGGGAGCGACC;  R: TCCAACTGTCACAACCTACTGG |
| *TNS1* | F: CACTCGCTGCCTTCTTCTGG;  R: TCTCAGTGGTGATGGGCACC |
| *FGF2* | F: AGTGTGTGCTAACCGTTACCT;  R: ACTGCCCAGTTCGTTTCAGTG |
| *PLAU* | F: GCAGCCACCGAGCCG;  R: CTGGTGCTGATCAGAGGTCC |
| *PRKCE* | F: CAACGGACGCAAGATCGAG;  R: CTGGCTCCAGATCAATCCAGT |
| *β-actin* | F: CACAGAGCCTCGCCTTTGCC;  R: ACCCATGCCCACCATCACG |

**Supplemental Table S3**

| Target gene | Site | Potential target sequence of 3'-UTR | Position of 3'-UTR |
| --- | --- | --- | --- |
| *ITGAV* | Site 1 | 5'. ..GUUUUAAAAAUGAUUGAAAUUUA... 3' | 1570–1577 |
|  | Site 2 | 5'. ..AUUUUUAUUACCAAUAAAUUUAA... 3' | 1802–1808 |
|  | Mutant Site 1 | 5'. ..GUUUUAAAAAUGAUU**CTTTAAA**A... 3' | 1570–1577 |
| *GAB1* | Site 1 | 5'. ..AGAAUCUUUUUAGAUGAAAUUUU... 3' | 2251–2257 |
|  | Site 2 | 5'. ..AUUUAAUCAUAGACUAAAUUUAA... 3' | 3122–3128 |
|  | Site 3 | 5'. ..CAAAAUCAUAUGUAUAAAUUUAU... 3' | 4024–4030 |
|  | Site 4 | 5'. ..CUGAUAAGGACAUAUAAAUUUAU... 3' | 4174–4180 |
|  | Site 5 | 5'. ..CAUACAGUGAUAUAGGAAAUUUA... 3' | 5175–5182 |
|  | Mutant Site 5 | 5'. ..CAUACAGUGAUAUAG**CTTTAAA**A... 3' | 5175–5182 |
| *TGFBR2* | Site 1 | 5'. ..UGACUUAAACCAACAGAAAUUUA... 3' | 7258–7265 |
|  | Site 2 | 5'. ..GGCUUCACUCUCUCUGAAAUUUU... 3' | 7329–7335 |
|  | Site 3 | 5'. ..UAAUUUUAUGGGUCAGAAAUUUG... 3' | 8163–8169 |
|  | Mutant Site 3 | 5'. ..UAAUUUUAUGGGUCA**CTTTAAA**G... 3' | 8163–8169 |
| *SRC* | Site 1 | 5'. ..GCAGUGCCUGCCUAUGAAAUUUC... 3' | 1703–1709 |
| *PPP2CB* | Site 1 | 5'. ..GCACAAUUUGAGACUGAAAUUUA... 3' | 189–196 |
|  | Site 2 | 5'. ..UUUUGCGCUUAUUUGGAAAUUUU... 3' | 317–323 |
