# Supplementary material for: miR-3606-3p alleviates skin fibrosis by integratively suppressing the integrin/FAK, p-AKT/p-ERK, and TGF-β signaling cascades
Source: J Adv Res. 2024 Nov 20;75:271–90. doi: 10.1016/j.jare.2024.11.027 (PMC12536609; doi:10.1016/j.jare.2024.11.027)
Supplement: Supplementary Data 4 [file mmc4.pdf]

CERTIFICATE

OF ENGLISH LANGUAGE EDITING

LetPub
EDITING CERTIFIED

miR-3606-3p alleviates skin fibrosis by integratively suppressing the integrin/FAK, p-AKT/p-ERK and TGF-β signaling cascades.

Introduction
Fibroblast abnormalities are crucial causes of skin fibrosis such as systemic sclerosis (SSc) and keloids. However, their mechanisms, including underlying microRNA regulatory mechanisms, remain elusive.
Objectives
This study aimed to evaluate the roles, mechanisms, and therapeutic potential of miR-3606-3p in regulating multiple fibroblast abnormalities.
Methods
The miR-3606-3p levels were evaluated in skin tissues and primary fibroblasts. RNA-seq and luciferase assays were employed to screen and validate miR-3606-3p targets. Collagen ...

This document certifies that the manuscript listed above was copy edited for English language by LetPub, with regard to grammar, punctuation, spelling, and clarity. Documents receiving this certification should be regarded as having undergone professional editorial revision for English language before submission. However, the authors may accept or reject LetPub's suggestions and changes at their own discretion and LetPub does not have editorial control over the submitted documents. Submitted documents may have new text that was not provided to LetPub for review. Please use the verification link below to determine the validity of the submitted version.

September 23, 2024

Date of Revision

LetPub

LetPub is an author service brand owned and operated by Accdon LLC.
Tel: 1-781-202-9968 Email: info@accdon.com
Address: 400 Fifth Ave, Suite 530, Waltham, MA 02451, United States

This manuscript has been individually edited for grammar, punctuation, spelling, and clarity. You may verify the authenticity of this certificate on our website (https://www.letpub.com/editorial-certificate) at any time using this manuscript's unique code: AS_240826X626.

© 2024 Accdon, LLC.
